# Hypoxia‐Induced O‐GlcNAcylation of GATA3 Leads to Excessive Testosterone Production in Preeclamptic Placentas

**DOI:** 10.1002/mco2.70115

**Published:** 2025-02-23

**Authors:** Juan Liu, Yun Yang, Hongyu Wu, Feihong Dang, Xin Yu, Feiyang Wang, Yongqing Wang, Yangyu Zhao, Xiaoming Shi, Wei Qin, Yanling Zhang, Yu‐Xia Li, Chu Wang, Xuan Shao, Yan‐Ling Wang

**Affiliations:** ^1^ State Key Laboratory of Stem Cell and Reproductive Biology Key Laboratory of Organ Regeneration and Reconstruction Institute of Zoology Chinese Academy of Sciences Beijing China; ^2^ Beijing Center for Disease Prevention and Control Beijing Key Laboratory of Diagnostic and Traceability Technologies for Food Poisoning Beijing China; ^3^ University of Chinese Academy of Sciences Beijing China; ^4^ Department of Obstetrics and Gynecology Peking University Third Hospital Beijing China; ^5^ Peking‐Tsinghua Center for Life Sciences Peking University Beijing China; ^6^ College of Chemistry and Molecular Engineering Peking University Beijing China; ^7^ Beijing Institute of Stem Cell and Regenerative Medicine Beijing China

**Keywords:** GATA3, hypoxia, O‐GlcNAcylation, preeclampsia, testosterone

## Abstract

The maintenance of endocrine homeostasis in the placenta is crucial for ensuring successful pregnancy. An abnormally elevated production of placental testosterone (T_0_) has been documented in patients with early‐onset preeclamptic (E‐PE). However, the underlying mechanisms remain unclear. In this study, we found that E‐PE placentas exhibited significantly increased expressions of 3β‐HSD1 (3β‐Hydroxysteroid Dehydrogenase 1) and 17β‐HSD3 (17β‐Hydroxysteroid Dehydrogenase 3), the rate‐limiting enzymes for T_0_ synthesis. This was strongly correlated with an elevated level of O‐linked N‐acetylglucosaminylation (O‐GlcNAcylation) of GATA3 (GATA binding protein 3). In human trophoblast cells, O‐linked‐N‐acetylglucosamine (O‐GlcNAc) modification of GATA3 on Thr^322^ stabilized the protein and enhanced the transcriptional regulation of 3β‐HSD1 and 17β‐HSD3, thereby increasing T_0_ production. Hypoxia, a well‐established pathological factor in PE, significantly enhanced the O‐GlcNAcylation of GATA3 in human trophoblast cells. Our findings suggest that hypoxia‐induced overactive O‐GlcNAcylation of GATA3 contributes to the exacerbated T_0_ production in E‐PE placentas. These findings provide a new perspective on the pathogenesis of E‐PE from the standpoint of posttranslational regulation and may illuminate novel therapeutic strategies for adverse pregnancy outcomes such as E‐PE.

## Graphical abstract

Hypoxia at the maternal–fetal interface of early‐onset preeclamptic placentas triggers global O‐GlcNAcylation. This process stabilizes the protein through O‐GlcNAcylation of GATA3 at Thr^322^ and transcriptionally induces the expression of 3β‐HSD1 and 17β‐HSD3, key androgen synthetases. Consequently, this accelerates the production of testosterone, thereby exacerbating the imbalance in the steroid hormones of patients with early‐onset preeclampsia.

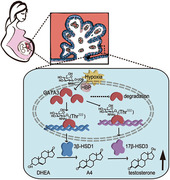

## 1 Introduction

The placenta is an important endocrine organ that synthesizes a variety of hormones throughout gestation to support fetal development and maternal adaptation to pregnancy [1]. In pregnant women, sex steroids such as progesterone, estrogen, and androgen are primarily produced by the placenta throughout gestation, with their levels increasing from the first trimester until the end of pregnancy [2]. The biosynthesis of these placental sex steroids begins with the conversion of cholesterol into pregnenolone by cytochrome P450 in the placenta, catalyzed by CYP17A1 to yield dehydroepiandrosterone (DHEA) in both maternal and fetal adrenal glands [3]. DHEA is then transported to the placenta for the synthesis of androstenedione (A_4_) and T_0_, with 3β‐HSD1 and 17β‐HSD3 acting as the primary synthases respectively. A_4_ and T_0_ are subsequently converted into estrogen by aromatase within the placenta [4].

Preeclampsia (PE), a pregnancy complication characterized by new‐onset hypertension, proteinuria, and multisystem disorders, is the leading cause of maternal and perinatal morbidity and mortality worldwide [5, 6, 7]. The etiology of preeclampsia remains elusive. However, disturbances in endocrine homeostasis are a significant pathological factor [4, 8, 9]. Our group and others have shown that patients with E‐PE (clinical symptoms appear before 34th gestational week) exhibit abnormally elevated levels of T_0_ while 17β‐estradiol (E_2_) levels are suppressed in the maternal circulation. This imbalance in hormone synthesis within the placenta accounts for these observations [10, 11]. When pregnant mice were administered the T_0_ analog testosterone propionate from E7.5 onwards, they developed PE‐like symptoms, accompanied by an increase in T_0_ levels and a decrease in E_2_ production [9]. This suggests that the abnormal surge in T_0_ synthesis is likely the primary cause of placental endocrine disturbances associated with PE [9]. However, the specific regulatory mechanism responsible for this remains to be elucidated.

Hypoxia at the maternal–fetal interface is a primary cause of PE [12]. This stress can markedly alter the O‐GlcNAc modification of proteins, instigating changes via the stimulation of the hexosamine biosynthetic pathway (HBP) flux [13, 14]. O‐GlcNAcylation, a reversible and ubiquitous posttranslational modification in eukaryotic cells, impacts a wide range of cellular processes [15, 16, 17]. Evidence has suggested the involvement of O‐GlcNAcylation in multiple events of pregnancy, including preimplantation embryo development [18], embryonic implantation [19], and placental response to stress [20]. The dysregulation of O‐GlcNAcylation resulting from maternal hyperglycemia impairs cell proliferation and blastocyst formation in preimplantation development [18]. Deletion of OGT (O‐GlcNAc transferase) in mouse placenta leads to early prenatal stress involving endocrine and immune signals, and dysregulation of the hypothalamic–pituitary–adrenal axis responsivity and function of hypothalamic mitochondrial in adult offsprings [21]. Such prenatal and long‐term offspring features strongly indicate the participation of O‐GlcNAcylation in maintaining intrauterine homeostasis. However, the relationship between protein O‐GlcNAcylation and the regulation of placental endocrine homeostasis remains unknown. We have recently developed a database of O‐GlcNAcylated proteins in human placental trophoblasts and demonstrated its role in modulating trophoblast differentiation via protein O‐GlcNAc modification [22]. Notably, GATA3 exhibits abundant O‐GlcNAc modifications within our database, aligning with existing literature that suggests GATA3 is uniquely enriched in the placenta compared to other tissues [23, 24]. Deletion of *Gata3* and *Gata2* genes in mouse trophoblasts resulted in compromised placental development and early embryonic mortality [25]. Furthermore, essential GATA‐binding sites for the transcriptional regulation of the human *HSD3B1* gene (encoding 3β‐HSD1) in the placenta have been identified [26]. Based on these findings, we hypothesized that hypoxic stress could intensify the O‐GlcNAc modification of GATA3 in placental trophoblasts, potentially promoting the overexpression of specific androgen synthases and consequently leading to excessive T_0_ production in E‐PE placentas.

This study examined the effect of O‐GlcNAcylation on GATA3 protein stability and transcriptional regulation of key steroid synthases 3β‐HSD1 and 17β‐HSD3 by GATA3, as well as T_0_ production in human trophoblast cells. We assessed the effect of hypoxic stress on GATA3 O‐GlcNAcylation and T_0_ production in trophoblasts, and the association between O‐GlcNAcylated GATA3 and 3β‐HSD1 and 17β‐HSD3 in E‐PE placentas. The aim was to elucidate the mechanism by which hypoxia‐induced increase in GATA3 O‐GlcNAcylation results in excessive androgen production in E‐PE placentas, thereby aiding in the identification of early etiological factors of endocrine disruption in E‐PE.

## 2 Results

### 2.1 A Strong Correlation Exists Between GATA3 and Androgen Synthetases, as Well as T_0_ Production in E‐PE Placentas

**FIGURE 1:**
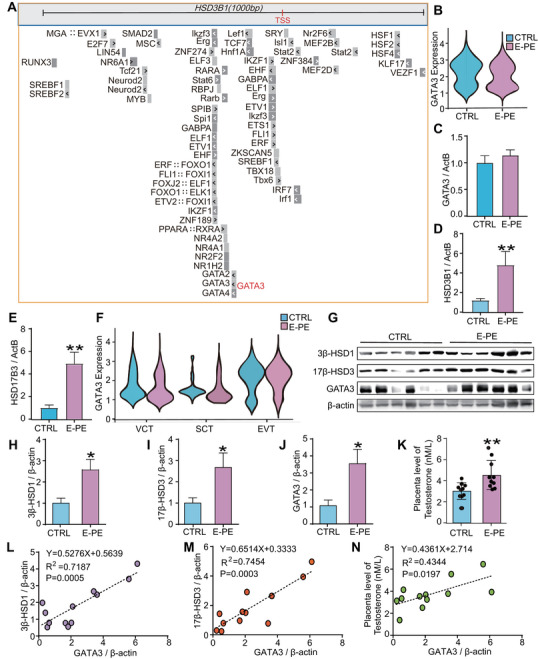
**The expression of GATA3, 3β‐HSD1, and 17β‐HSD3, along with testosterone (T_0_) production in early‐onset preeclampsia (E‐PE) placentas**. (**A**) The hg38 genome was selected from the JASPAR transcription factor database via the UCSC database (http://genome.ucsc.edu/). Transcription factors for target genes were predicted 1 kb upstream of the gene's transcription start site (TSS). The prediction process employed a minimum score threshold of 400 (*p* < 1 × 10^−4^) to ensure accuracy. (**B**) The mRNA expression of *GATA3* in E‐PE and gestational age‐matched preterm labor (CTRL) placentas was examined through single‐cell transcriptome sequencing. (**C**) mRNA expression of *GATA3* in E‐PE (*n* = 6) and CTRL placentas (*n* = 6). (**D**, **E**) mRNA level of *HSD3B1* (**D**) and *HSD17B3* (**E**) in E‐PE (*n* = 6) and CTRL placentas (*n* = 6). (**F**) The mRNA expression of *GATA3* in trophoblasts of the normal human placenta was examined through single‐cell transcriptome sequencing. VCT, villous cytotrophoblast; SCT, syncytiotrophoblast; EVT, extravillous trophoblast. (**G**–**J**) Western blotting (**G**) and statistical analysis (**H**–**J**) of 3β‐HSD1, 17β‐HSD3, and GATA3 levels in E‐PE (*n* = 6) and CTRL placentas (*n* = 6). (**K**) Testosterone (T_0_) level in E‐PE (*n* = 6) and CTRL placentas (*n* = 6) determined by immunoassay. (**L‐**‐**N**) Correlation between GATA3 and 3β‐HSD1 (**L**), 17β‐HSD3 (**M**), or T_0_ levels (**N**) in E‐PE and CTRL placentas. Data are shown as the mean ± SEM from at least three independent experiments and were analyzed by a two‐tailed *t*‐test (**C–E**, **H–K**). Nonparametric Spearman's correlation test was used to analyze the correlations among 3β‐HSD1, 17β‐HSD3, or T_0_ and GATA3 levels (**L**–**N**). *p < 0.05; **p < 0.01.

To identify the transcription factors that regulate 3β‐HSD1 and 17β‐HSD3 expression, we first examined the motif composition of the sequence upstream of the transcription start site (TSS) of *HSD3B1* and *HSD17B3*. We found a high frequency of potential transcription factor binding motifs in the proximal promoter region of *HSD3B1* (Figure 1A). Among these factors, GATA3 was specifically enriched in placenta compared to other tissues [23, 24]. GATA3 can promote extraembryonic trophoblasts to adopt their definitive fate at the blastocyst stage and stimulate trophoblast stem cells differentiation [27]. A GATA3 binding motif was also identified within the gene region for *HSD17B3* (encoding 17β‐HSD3) by ChIP‐seq analysis conducted on MCF7 cells and human embryonic stem cell derived trophoblast progenitors (GSE51274, GSE122847, GSE40129, and GSE105081; Figure S1). According to the single‐cell transcriptome sequencing [28] and RT‐qPCR results, we found that *GATA3* was expressed at relatively high level in the human placenta and trophoblasts (Figures S2 and S3). Surprisingly, *GATA3* transcriptional expression was similar between E‐PE and the CTRL (Figure 1B, C). In contrast, mRNA levels of *HSD3B1* and *HSD17B3* were significantly higher in E‐PE placentas (Figure 1D, E). Considering the heterogeneous composition of the placenta, we next investigated *GATA3* expression by trophoblast cell type in human placenta. We confirmed that *GATA3* expression is similar between E‐PE and CTRL regardless of cell type (Figure 1F).

Interestingly, although the mRNA level of *GATA3* was not altered, its protein level was markedly upregulated in E‐PE placentas, accompanied by increased 3β‐HSD1, 17β‐HSD3 and T_0_ levels (Figures 1G–K and S4). The nonparametric Spearman's correlation test showed a strong positive correlation between the level of GATA3 and 3β‐HSD1, 17β‐HSD3 or T_0_ (Figure 1L–N). These results indicate that the GATA3 protein may be regulated by posttranslational modifications, which may further contribute to the excessive production of T_0_ in E‐PE placentas.

### 2.2 O‐GlcNAcylation Dynamically Modified and Stabilized GATA3 Protein in Human Trophoblast Cells

**FIGURE 2:**
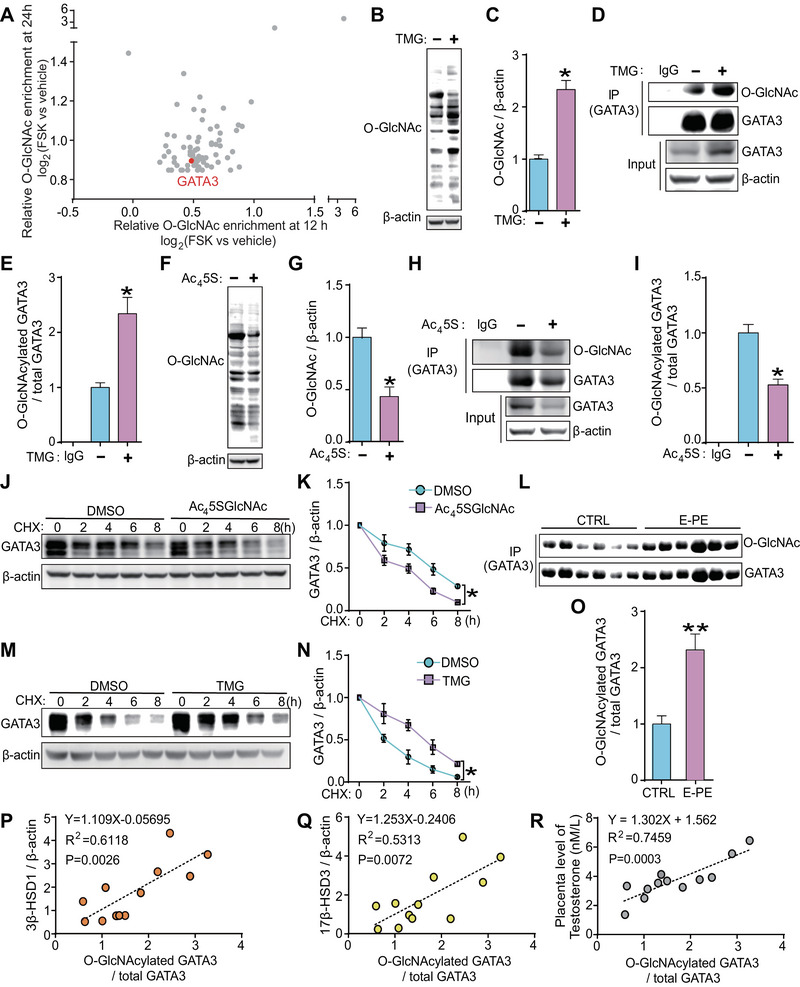
**O‐GlcNAcylation dynamically modified and stabilized GATA3 protein in human trophoblast cells**. (**A**) Scatter plot illustrating the ratios of O‐GlcNAcylated proteins in response to 20 µM forskolin (FSK) at 12 and 24 h. Gray points indicate the logarithmic values of upregulation of intracellular O‐GlcNAcylated proteins at 12 and 24 h (H/L > 1.8). Red points indicate the ratios of O‐GlcNAcylated GATA3. (**B**, **C**) Western blotting (**B**) and statistical analysis (**C**) of total O‐GlcNAcylated proteins in JEG3 cells treated with 20 µM thiamet‐G (TMG). (**D**, **E**) Immunoblotting following immunoprecipitation (IP) (**D**) and statistical analysis (**E**) of O‐GlcNAcylated GATA3 in JEG3 cells treated with 20 µM TMG. (**F**, **G**) Western blotting (**F**) and statistical analysis (**G**) of total O‐GlcNAcylated proteins in JEG3 cells treated with 20 µM Ac_4_5S. (**H**, **I**) Immunoblotting following IP (**H**) and statistical analysis (**I**) of O‐GlcNAcylated GATA3 in JEG3 cells treated with 20 µM Ac_4_5S. (**J**, **K**) Western blotting (**J**) and statistical analysis (**K**) of GATA3 in JEG3 cells treated with 20 µM Ac_4_5S, and then treated with cycloheximide (CHX) for varying durations. (**L**, **M**) Western blotting (**L**) and statistical analysis (**M**) of GATA3 in JEG3 cells treated with 20 µM TMG, and then treated with CHX for varying durations. (**N**–**O**) Immunoblotting following IP (**N**) and statistical analysis (**O**) of O‐GlcNAcylated GATA3 in E‐PE (*n* = 6) and CTRL placentas (*n* = 6). (**P**–**R**) Correlation between the relative O‐GlcNAc stoichiometry of GATA3 and 3β‐HSD1 (**P**), 17β‐HSD3 (**Q**), or T_0_ levels (**R**) in E‐PE and CTRL placentas. Data are shown as the mean ± SEM of at least three independent experiments and were analyzed by two‐tailed *t*‐test (**C**, **G**, **O**) or one‐way ANOVA and Tukey–Kramer multiple comparison test (**E**, **I**, **K**, **M**). Nonparametric Spearman's correlation test was used to analyze the correlations among 3β‐HSD1, 17β‐HSD3, or T_0_ and the relative O‐GlcNAc stoichiometry of GATA3 (**P–** **R**). *p < 0.05; **p < 0.01.

Among the proteins in our human placental trophoblasts O‐GlcNAcylated protein database [22], GATA3 was identified as one of the most heavily O‐GlcNAc modified proteins (Figure 2A). To investigate whether GATA3 can be O‐GlcNAcylated, we quantified the changes in O‐GlcNAcylated GATA3 levels in JEG3 cells treated with Thiamet G (TMG, an inhibitor of O‐GlcNAcase) or Ac_4_5SGlcNAc (Ac_4_5S, an inhibitor of O‐GlcNAc transferase). As expected, TMG treatment significantly increased global O‐GlcNAcylation levels (Figure 2B, C) and the relative O‐GlcNAc stoichiometry of GATA3 (Figure 2D, E). Conversely, Ac_4_5S led to a significant decrease in global O‐GlcNAcylation (Figure 2F, G) and O‐GlcNAcylated GATA3 levels (Figure 2H, I). These results clearly demonstrate that GATA3 can be dynamically O‐GlcNAcylated in human trophoblast cells.

Since TMG or Ac_4_5S exposure in JEG3 cells resulted in alterations in GATA3 protein but not mRNA expression (Figures 2D, E, H, I and S5), we hypothesized that O‐GlcNAc modification could stabilize GATA3 protein. Accordingly, JEG3 cells were treated with Ac_4_5S or TMG for 24 h, followed by treatment with the protein synthesis inhibitor cycloheximide (CHX) for 0–8 h. The time‐dependent decrease of GATA3 protein levels after CHX treatment was significantly accelerated upon O‐GlcNAc deprivation with Ac_4_5S (Figure 2J, K), whereas it was considerably retarded upon O‐GlcNAc augmentation by TMG, compared to the respective controls at different time points (Figure 2L, M). Furthermore, IP experiments revealed a higher O‐GlcNAc stoichiometry of GATA3 (O‐GlcNAcylated GATA3 / total GATA3) in E‐PE placentas, which was 2.2‐fold higher than that in CTRL placentas (Figure 2N, O). A nonparametric Spearman's correlation test showed a strong positive correlation between the levels of O‐GlcNAcylated GATA3 and 3β‐HSD1, 17β‐HSD3, or T_0_ (Figure 2P–R). These results clearly demonstrate that O‐GlcNAcylation regulates GATA3 protein stability and suggest that the increased T_0_ levels in E‐PE placentas might be regulated by O‐GlcNAcylated GATA3.

### 2.3 Thr^322^ Is the Primary O‐GlcNAcylation Site for GATA3 in Human Trophoblast Cells

**FIGURE 3:**
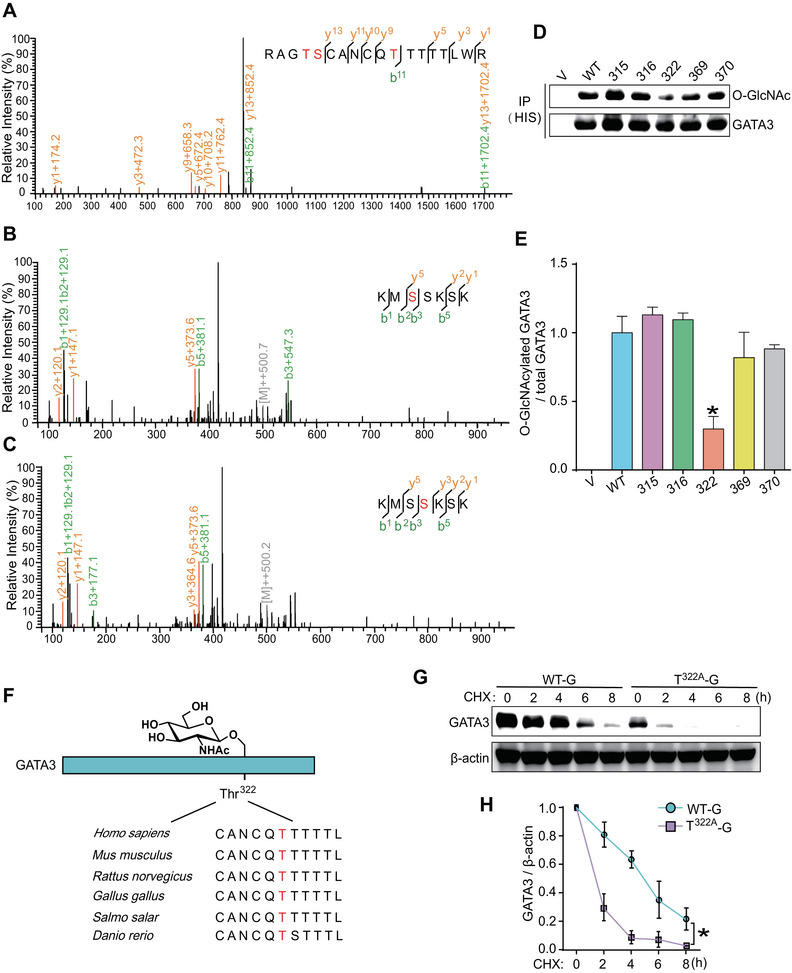
**Thr^322^ is the primary O‐GlcNAcylation site for GATA3 in human trophoblast cells**. (**A‐**‐**C**) HCD mass spectrum of the peptide on GATA3 containing the O‐GlcNAc modification at Thr^315^, Ser^316^, Thr^322^ (**A**), Ser^369^ (**B**), and Ser^370^ (**C**). The matched fragment y and b ions are labeled in orange and green, respectively; O‐GlcNAcylated sites are labeled in red. (**D**, **E**) Immunoblotting following IP (**D**) and statistical analysis (**E**) of O‐GlcNAcylated GATA3 in JEG3 cells transfected with WT‐G (WT), T^315A^‐G (315), S^316A^‐G (316), T^322A^‐G (322), S^369A^‐G (369), and S^370A^‐G (370) plasmid. (**F**) Sequence alignment of peptide containing Thr^322^ of human GATA3 and corresponding regions from other vertebrate orthologs. (**G**–**H**) Western blotting (**G**) and statistical analysis (**H**) of GATA3 in JEG3 cells transfected with either WT‐G or T^322A^‐G plasmid, and then treated with CHX for varying durations. Data are shown as the mean ± SEM of at least three independent experiments and were analyzed by one‐way ANOVA and Tukey–Kramer multiple comparison test. *p < 0.05.

To identify the O‐GlcNAcylation site(s) of GATA3 in trophoblasts, we conducted an IP using an anti‐GATA3 antibody and subjected the in‐gel trypsin digested peptides to LC–MS/MS analysis as previously described [29]. This process revealed five putative O‐GlcNAcylation sites (Thr^315^, Ser^316^, Thr^322^, Ser^369^, and Ser^370^) within GATA3 (Figure 3A–C). We generated five distinct plasmids that separately carried GATA3 with one of these putative O‐GlcNAcylation residues mutated to valine (T^315A^‐G, S^316A^‐G, T^322A^‐G, S^369A^‐G, and S^370A^‐G), and transfected them into JEG3 cells. The O‐GlcNAcylation levels of GATA3 in these cells were then measured following TMG exposure. Notably, T^322A^‐G significantly reduced the relative O‐GlcNAc stoichiometry of GATA3 compared to other plasmids (Figure 3D, E). A sequence comparison between *Homo sapiens* and other vertebrate orthologs revealed a high degree of conservation of the GATA3‐Thr^322^ residue among mammals (Figure 3F). These findings collectively demonstrate that Thr^322^ is the primary O‐GlcNAcylation site for GATA3 in human trophoblast cells. Consequently, JEG3 cells transfected with the T^322A^‐G plasmid exhibited a significantly faster degradation of GATA3 than cells transfected with the WT‐G plasmid (Figure 3G, H). Therefore, the stability of GATA3 can be directly modulated by O‐GlcNAcylation at Thr^322^.

### 2.4 GATA3 Transcriptionally Regulated the Expression of 3β‐HSD1 and 17β‐HSD3 in Human Trophoblast Cells

**FIGURE 4:**
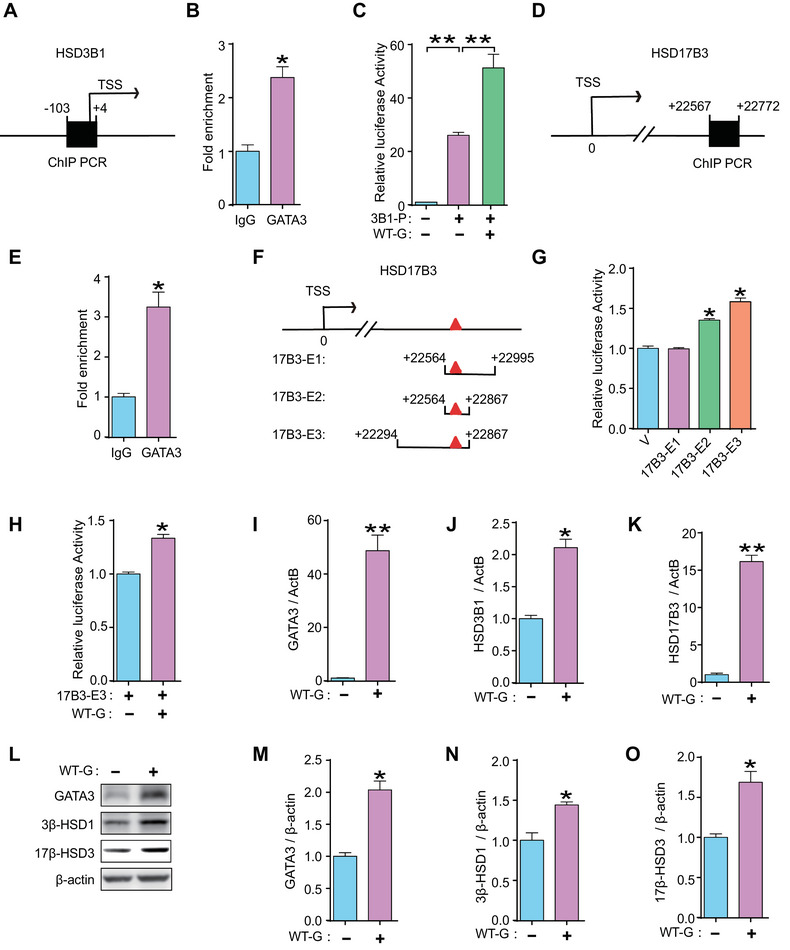
**GATA3 transcriptionally regulated the expression of 3β‐HSD1 and 17β‐HSD3 in human trophoblast cells**. (**A**) Schematic representation of the promoter region for the *HSD3B1* gene. Nucleotide locations are indicated by numbers, with 0 denoting the TSS. (**B**) ChIP analysis in JEG3 cells to examine the binding of GATA3 to the promoter region of the *HSD3B1* gene. (**C**) Dual‐luciferase assay in JEG3 cells transfected with plasmid carrying the promoter region of *HSD3B1* (3B1‐P) with or without the inclusion of a plasmid expressing GATA3 (WT‐G). (**D**) Schematic representation of the predicted GATA binding region in *HSD17B3* gene locus. Nucleotide locations are indicated by numbers, with 0 denoting the TSS. (**E**) ChIP analysis in JEG3 cells to examine the binding of GATA3 to the predicted GATA‐binding region of *HSD17B3*. (**F**) Schematic representation of the plasmids (17B3‐E1, 17B3‐E2, 17B3‐E3), each containing distinct regions of the predicted GATA‐binding region (red triangle) of *HSD17B3*. (**G**) Dual‐luciferase assay in JEG3 cells transfected with 17B3‐E1, ‐E2, or ‐E3 plasmid. (**H**) Dual‐luciferase assay in JEG3 cells transfected with 17B3‐E3 with or without WT‐G plasmid. (**I**–**K**) mRNA level of *GATA3* (**I**), *HSD3B1* (**J**), and *HSD17B3* (**K**) in JEG3 cells transfected with WT‐G plasmid. (**L**–**O**) Western blotting (**L**) and statistical analysis (**M–** **O**) of GATA3, 3β‐HSD1, and 17β‐HSD3 in JEG3 cells transfected with WT‐G plasmid. Data are shown as the mean ± SEM of at least three independent experiments and were analyzed by two‐tailed *t*‐test (**B**, **E**, **H–** **K**, **M**–**O**) or one‐way ANOVA and Tukey–Kramer multiple comparison test (**C**, **G**). *p < 0.05; **p < 0.01.

To determine the interaction between GATA3 and *HSD3B1* in human placental trophoblasts, ChIP experiments were performed using JEG3 cells. We found that GATA3 bound significantly to the promoter region of *HSD3B1* (Figure 4A, B). A luciferase reporter plasmid containing the *HSD3B1* promoter region (3B1‐P) was constructed and co‐transfected with a GATA3‐expressing plasmid (WT‐G) into JEG3 cells. The dual‐luciferase assay showed that GATA3 markedly enhanced the activity of the *HSD3B1* promoter (Figure 4C), indicating strong transcriptional regulation of *HSD3B1* by GATA3 in human trophoblast cells.

Data mining of the ChIP‐seq database (GSE105081) revealed a putative GATA‐binding motif approximately 22 kb downstream of the TSS in *HSD17B3* (Figure S1). Further ChIP analyses confirmed significant binding of GATA3 to this motif in *HSD17B3* (Figure 4D, E). Three plasmids (17B3‐E1, ‐E2, and ‐E3) containing different regions of the predicted GATA‐binding motif in *HSD17B3* were constructed (Figure 4F) and transfected separately into JEG3 cells with or without the WT‐G plasmid. The highest luciferase activity was observed upon transfection with the 17B3‐E3 plasmid (Figure 4G, H), suggesting enhancer activity of GATA3 on *HSD17B3* expression.

Moreover, GATA3‐overexpressed JEG3 cells showed a marked upregulation of *HSD3B1* and *HSD17B3* mRNA levels and increased protein levels of 3β‐HSD1 and 17β‐HSD3 (Figure 4I–O). Single‐cell ATAC‐seq (assay for transposase‐accessible chromatin using sequencing) analysis demonstrated that the *HSD3B1* and *HSD17B3* gene loci are accessible in trophoblast linages, such as VCT (villous cytotrophoblast), SCT (syncytiotrophoblast), and EVT (extravillous trophoblast), which is accompanied by an elevated expression of GATA3 in these cell types, compared to dNK (decidual natural killer cell), a major population of non‐trophoblast cells at the maternal–fetal interface [30] (Figure S6). Moreover, there was no effect on the proliferation of JEG3 cells after GATA3 overexpression (Figure S7).

Collectively, these results show that GATA3 can transcriptionally upregulate 3β‐HSD1 and 17β‐HSD3 expression in human trophoblasts.

### 2.5 O‐GlcNAcylation of GATA3 Markedly Enhanced T_0_ Synthesis in Human Trophoblast Cells

**FIGURE 5:**
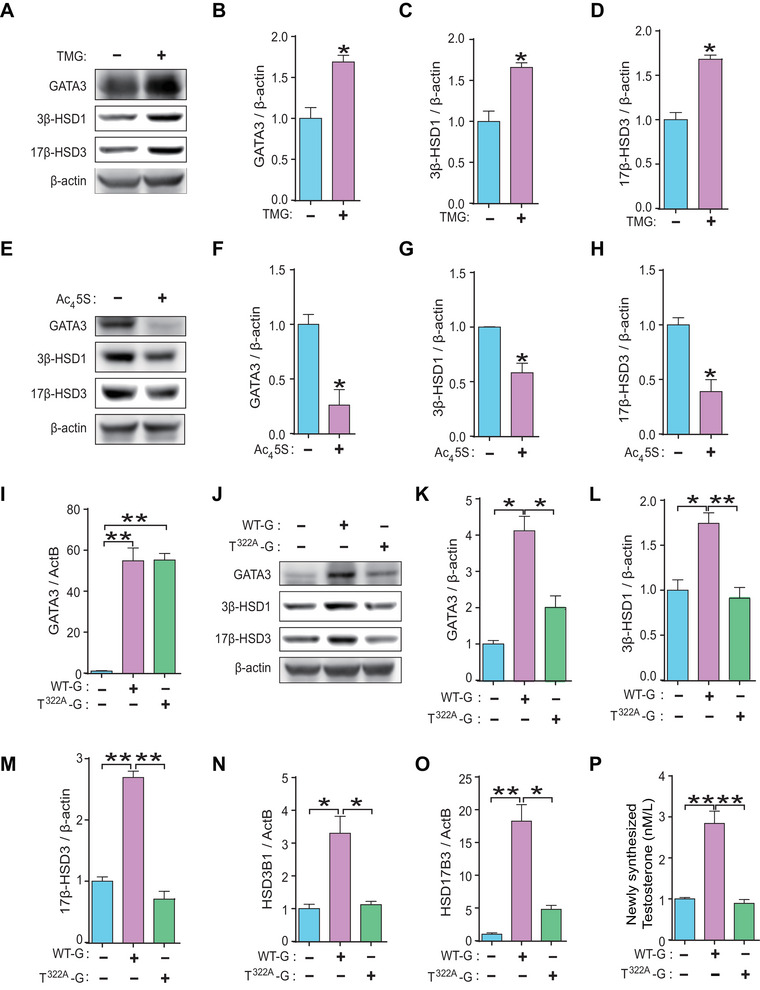
**O‐GlcNAcylation of GATA3 markedly enhanced T_0_ synthesis in human trophoblast cells**. (**A**–**D**) Western blotting (**A**) and statistical analysis (**B**–**D**) of GATA3, 3β‐HSD1, and 17β‐HSD3 in JEG3 cells treated with 20 µM TMG. (**E–** **H**) Western blotting (**E**) and statistical analysis (**F**–**H**) of GATA3, 3β‐HSD1, and 17β‐HSD3 in JEG3 cells treated with 20 µM Ac_4_5S. (**I**) mRNA level of *GATA3* in JEG3 cells transfected with WT‐G or T^322A^‐G plasmids. (**J–** **M**) Western blotting (**J**) and statistical analysis (**K** **–M**) of GATA3, 3β‐HSD1, and 17β‐HSD3 in JEG3 cells transfected with WT‐G or T^322A^‐G plasmids. (**N**, **O**) mRNA level of *HSD3B1* (**N**) and *HSD17B3* (**O**) in JEG3 cells transfected with WT‐G or T^322A^‐G plasmids. (**P**) Newly synthesized T_0_ level in the culture supernatant of JEG3 cells transfected with WT‐G or T^322A^‐G plasmids. Data are shown as the mean ± SEM of at least three independent experiments and were analyzed by two‐tailed *t*‐test (**B–** **D**, **F**–**H**) or one‐way ANOVA and Tukey–Kramer multiple comparison test (**I**, **K–** **P**). *p < 0.05; **p < 0.01.

To examine the role of GATA3 O‐GlcNAcylation in T_0_ synthesis within human trophoblasts, JEG3 cells were subjected to treatment with TMG or Ac_4_5S, or transfected with either T^322A^‐G or WT‐G plasmids. As anticipated, levels of GATA3, 3β‐HSD1 and 17β‐HSD3 were significantly elevated in cells treated with TMG (Figure 5A–D). Conversely, these protein levels were notably reduced in cells treated with Ac_4_5S (Figure 5E–H). The mRNA level of *GATA3* was significantly upregulated upon transfection with either the T^322A^‐G or the WT‐G plasmid, with no significant differences observed between these groups (Figure 5I). However, while transfection with the WT‐G plasmid resulted in a threefold increase in GATA3 protein expression, transfection with the T^322A^‐G plasmid led to a slight but non‐significant increase in GATA3 protein expression compared to control cells (Figure 5J, K). Furthermore, overexpression of GATA3 by the WT‐G plasmid significantly enhanced the expressions of 3β‐HSD1 and 17β‐HSD3 as well as T_0_ production, whereas these effects were completely abolished in cells transfected with T^322A^‐G (Figure 5J, L–P). Taken together, these findings demonstrate that O‐GlcNAc modification of GATA3 effectively enhances T_0_ synthesis by stimulating the expression of key androgen synthases in human trophoblast cells.

### 2.6 Hypoxic Stress Induced the O‐GlcNAcylation of GATA3 and Led to an Overproduction of T_0_ Production in Human Trophoblast Cells

**FIGURE 6:**
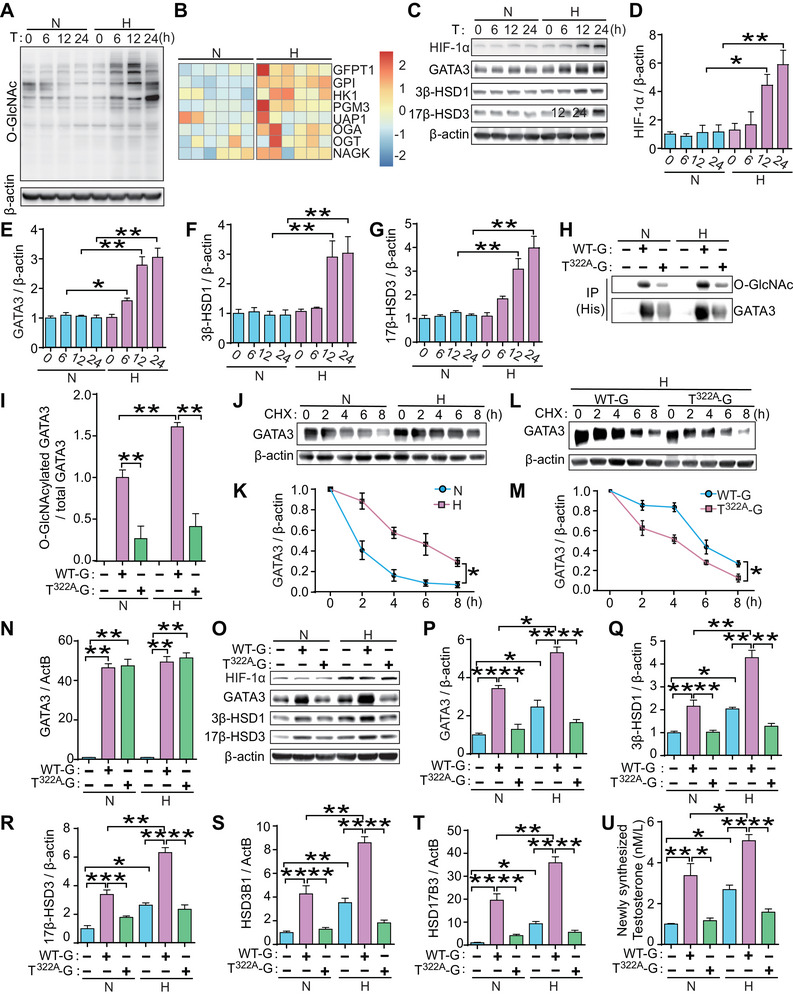
**Hypoxic stress induced the O‐GlcNAcylation of GATA3 and led to an overproduction of T_0_ in human trophoblast cells**. (**A**) Western blotting of whole O‐GlcNAcylated protein in JEG3 cells exposed to either 21% or 2% O_2_. (**B**) A heatmap of the transcriptome gene, specifically those directly associated with enzymes in HBP dand O‐GlcNAc cycling, was generated from a single‐cell analysis of human trophoblast stem cells. These cells were exposed to either 21% or 2% O_2_ for a duration of four days (*n* = 6). The visualization of this heatmap was based on the Gene Transcription Levels Metric, with the display being contingent upon the Z score of gene expression. (**C‐**‐**G**) Western blotting (**C**) and statistical analysis (**D‐**‐**G**) of HIF‐1α, GATA3, 3β‐HSD1, and 17β‐HSD3 in JEG3 cells exposed to either 21% or 2% O_2_, supplemented with 20 µM MG132 (an inhibitor of proteasome and calpain). (**H**, **I**) Immunoblotting following IP (**H**) and statistical analysis (**I**) of O‐GlcNAcylated GATA3 in JEG3 cells transfected with WT‐G or T^322A^‐G plasmids under either 21% or 2% O_2_. (**J**, **K**) Western blotting (**J**) and statistical analysis (**K**) of GATA3 in JEG3 cells exposed to either 21% or 2% O_2_, and then treated with CHX for varying durations. (**L**, **M**) Western blotting (**L**) and statistical analysis (**M**) of GATA3 in JEG3 cells transfected with either WT‐G or T^322A^‐G plasmid under 2% O_2_, and then treated with CHX for varying durations. (**N**) mRNA level of *GATA3* in JEG3 cells transfected with WT‐G or T^322A^‐G plasmids under either 21% or 2% O_2_. (**O**–**R**) Western blotting (**O**) and statistical analysis (**P**–**R**) of GATA3, 3β‐HSD1, and 17β‐HSD3 in JEG3 cells transfected with WT‐G or T^322A^‐G plasmids under either 21% or 2% O_2_, supplemented with 20 µM MG132. (**S**, **T**) mRNA level of *HSD3B1* (**S**) and *HSD17B3* (**T**) in JEG3 cells transfected with WT‐G or T^322A^‐G plasmids under either 21% or 2% O_2_. (**U**) Newly synthesized T_0_ level in the culture supernatant of JEG3 cells transfected with WT‐G or T^322A^‐G plasmids under either 21% or 2% O_2_. N refers to 21% O_2_ or normoxia, while H refers to 2% O_2_ or hypoxia. Data are shown as the mean ± SEM of at least three independent experiments and were analyzed by one‐way ANOVA and Tukey–Kramer multiple comparison test. *p < 0.05; **p < 0.01.

To elucidate the pathological mechanism underlying T_0_ overproduction in E‐PE placentas, we investigated whether hypoxic stress induces GATA3 O‐GlcNAcylation in trophoblasts. JEG3 cells were exposed to 2% O_2_ for different time periods (0–24 h) to mimic the hypoxic environment in PE placentas [31]. The global O‐GlcNAcylation level was increased after exposure to hypoxia for 6–24 h (Figure 6A). From the RNA‐seq data (GSE159480) [32] of hypoxia‐exposed placental iPSCs differentiated into syncytiotrophoblast, we analyzed the enzymes directly related to HBP and O‐GlcNAc cycling. We found that expression levels of almost all enzymes were higher than those in the control group, indicating that HBP was activated following hypoxia stimulation (Figure 6B). This result is consistent with previous reports showing that cellular stress, such as hypoxia, significantly alters the O‐GlcNAc modification of proteins by stimulating HBP flux [13, 14]. Hypoxia exposure dramatically increased the levels of HIF‐1α, GATA3, 3β‐HSD1, and 17β‐HSD3 compared to their corresponding normoxia group (21% O_2_) (Figure 6C–G). IP experiments showed that the relative O‐GlcNAcylation level of GATA3 in WT‐G‐transfected JEG3 cells was significantly increased by hypoxia, whereas mutations in Thr^322^ reduced this effect (Figure 6H, I). Moreover, the time‐dependent degradation of GATA3 was slower in hypoxia‐exposed JEG3 cells than in normoxic cultures (Figure 6J, K). However, the hypoxia‐induced degradation of GATA3 was significantly weakened by the mutation in Thr^322A^, as shown by a comparison between WT‐G‐ and Thr^322A^‐G‐transfected cells under hypoxic stimulation (Figure 6L, M). These results confirm that hypoxic stress in human trophoblast cells promotes GATA3 O‐GlcNAcylation, resulting in enhanced protein stability.

To further confirm whether hypoxia‐induced GATA3 O‐GlcNAcylation contributes to T_0_ overproduction, we analyzed WT‐G‐ or T^322^‐G‐transfected JEG3 cells under 2% or 21% O_2_. The mRNA level of *GATA3* was similar between the WT‐G and T^322^‐G groups and was not altered by hypoxic stimulation (Figure 6N). However, hypoxia exposure significantly increased the protein levels of GATA3, 3β‐HSD1, and 17β‐HSD3 (Figure 6O–R), the mRNA expression of *HSD3B1* and *HSD17B3* (Figure 6S, T), and T_0_ production (Figure 6U) in either basal or WT‐G‐overexpressing JEG3 cells when compared with their respective normoxic controls. In contrast, the stimulatory effects of hypoxia on these molecules and T_0_ production were abrogated in T^322A^‐G‐transfected cells (Figure 6O–U). These results clearly demonstrate that hypoxia induces an overproduction of T_0_ in trophoblasts via the induction of persistent GATA3 O‐GlcNAcylation, which strongly enhances the transcription of T_0_ synthase genes.

### 2.7 O‐GlcNAcylation of GATA3 Markedly Enhanced T_0_ Synthesis in Primary Human Trophoblast (PHT) Cells

**FIGURE 7:**
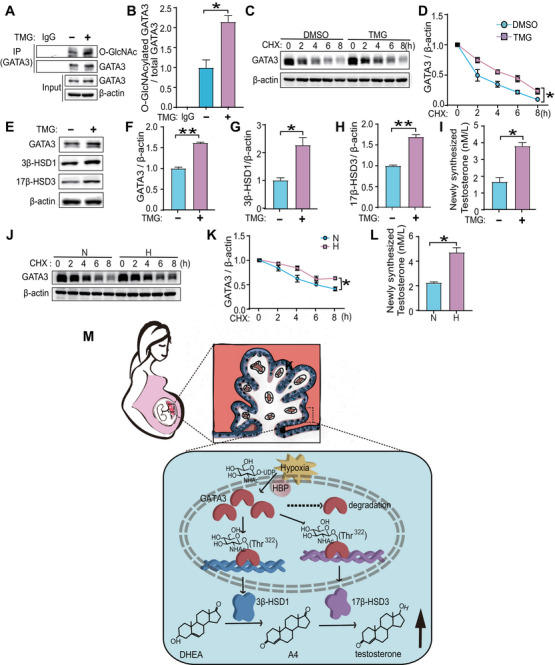
**O‐GlcNAcylation of GATA3 marketed enhanced T_0_ synthesis in primary human trophoblast (PHT) cells**. (**A**, **B**) Immunoblotting following IP (**A**) and statistical analysis (**B**) of O‐GlcNAcylated GATA3 in PHT cells treated with 20 µM TMG. (**C**, **D**) Western blotting (**C**) and statistical analysis (**D**) of GATA3 in PHT cells treated with 20 µM TMG, and then treated with CHX for varying durations. (**E‐**‐**H**) Western blotting (**E**) and statistical analysis (**F‐**‐**H**) of GATA3 (**F**), 3β‐HSD1 (**G**), and 17β‐HSD3 (**H**) in PHT cells treated with 20 µM TMG. (**I**) Newly synthesized T_0_ level in the culture supernatant of PHT cells treated with 20 µM TMG. (**J**, **K**) Western blotting (**J**) and statistical analysis (**K**) of GATA3 in PHT cells exposed to either 21% or 2% O_2_ for 24 h, and then treated with CHX for varying durations. (**L**) Newly synthesized T_0_ level in the culture supernatant of PHT cells exposed to either 21% or 2% O_2_ for 24 h. (**M**) Working model of hypoxia‐induced GATA3 O‐GlcNAcylation exacerbating T_0_ production in E‐PE placentas. Hypoxia at the maternal–fetal interface of E‐PE placentas triggers global O‐GlcNAcylation. This process stabilizes the protein through O‐GlcNAcylation of GATA3 at Thr^322^ and transcriptionally stimulates the expression of 3β‐HSD1 and 17β‐HSD3. As a result, this process accelerates the production of T_0_, thereby exacerbating the imbalance in the steroid hormones among patients with E‐PE. Data are shown as the mean ± SEM of at least three independent experiments and were analyzed by two‐tailed *t*‐test. *p < 0.05; **p < 0.01.

To further validate our findings, we next performed a series of validation experiments using PHT cells. As expected, TMG treatment markedly increased the relative O‐GlcNAc stoichiometry of GATA3 (Figure 7A, B). Furthermore, the time‐dependent decrease of GATA3 protein levels after CHX treatment was significantly retarded upon O‐GlcNAc augmentation by TMG compared to their respective controls at different time points in PHT cells (Figure 7C, D). In addition, PHT cells were treated with TMG or DMSO and as expected, GATA3, 3β‐HSD1 and 17β‐HSD3 levels and newly synthesized T_0_ were significantly elevated in TMG‐treated cells (Figure 7E–I). Furthermore, the time‐dependent degradation of GATA3 was markedly slower in hypoxia‐exposed PHT cells than in normoxic cultures (Figure 7J, K). Notably, exposure to hypoxia significantly enhanced T_0_ production compared to normoxia group (Figure 7L). Taken together, these results in PHT cells provide additional evidence for our hypothesis that hypoxia and O‐GlcNAcylation induced stabilization of GATA3 contributes to excessive T_0_ production.

## 3 Discussion

Our study, along with others, has identified a significant endocrine disruption in estrogen and androgen synthesis, particularly evident in E‐PE [10, 33]. Steroid hormones, mainly produced by the placenta during gestation, are pivotal for normal pregnancy [33]. Preeclampsia, a multisystem complication defined as newly onset hypertension and proteinuria in pregnancy that cause maternal and perinatal morbidity and mortality, commonly manifests with significantly high plasma testosterone (T_0_) level [4, 8, 9]. However, the underlying mechanism remains unclear. Here, we discovered that the expression of 3β‐HSD1 and 17β‐HSD3, key enzymes responsible for T_0_ synthesis, were significantly increased in E‐PE placentas, together with elevated level of O‐GlcNAcylated GATA3. A positive correlation existed between the expression of O‐GlcNAcylated GATA3 and placental T_0_ concentration. In human trophoblast cells, dynamic O‐GlcNAcylation modification of Thr^322^ stabilized GATA3, which empowered GATA3 to transcriptionally upregulate the expression of 3β‐HSD1 and 17β‐HSD3, and thus elevated the production of T_0_. As a well‐recognized pathological factor in PE, hypoxia can significantly enhance the O‐GlcNAcylation of GATA3 in human trophoblast cells (Figure 7M). The findings reveal the underlying mechanism that exacerbated high T_0_ production modulated by O‐GlcNAcylated GATA3 in E‐PE placentas and provide new insights into the pathogenesis of E‐PE from the perspective of endocrine homeostasis regulation.

Our previous study showed that androgen overproduction could inhibit estrogen production through the miR‐22/ERα/aromatase pathway in PE placentas [10]. In addition, testosterone propionate administration to pregnant mice induced hypertension and proteinuria, fetal and placental growth retardation, and E_2_ production impairment—phenotypes similar to those of E‐PE [9]. These findings suggest that abnormally high levels of T_0_ synthesis are a major cause of placental endocrine disorders in E‐PE, but the underlying mechanisms remain unclear. The present study provides a new insight into the pathogenesis of E‐PE by suggesting that persistent O‐GlcNAcylation induced by hypoxia regulates GATA3 stability, thereby increasing 3β‐HSD1 and 17β‐HSD3 levels transcriptionally and promoting excessive T_0_ synthesis in human trophoblast cells. It is well known that persistent hypoxic stress at the maternal–fetal interface contributes to suboptimal remodeling of the uterine spiral arteries in PE [12, 31]. During the normal course of pregnancy, uterine spiral artery remodeling begins around 7th to 8th week [34] and is completed by 20–22 weeks [35]. Our previous study showed that imbalanced estrogen and androgen synthesis was first observed in patients at 11–14 weeks of gestation [11]. Taken together with our findings that hypoxia promotes T_0_ synthesis, it can be speculated that the dysfunction of spiral artery remodeling at the maternal–fetal interface in E‐PE occurs early in pregnancy. Thus, this study not only reveals the pathogenesis of endocrine disorder in E‐PE but also provides a rationale for determining the developmental period of disturbance in E‐PE placentas. However, further studies are required to confirm this hypothesis.

GATA is a pioneer transcription factor that plays an important role in embryonic development [36]. Among the GATA family members, GATA3 has the highest expression in the placenta [23]. Deletion of GATA3 in the placenta leads to impaired placental development and fetal death [25]. Several studies have shown that GATA3 plays an essential role in trophoblast cell differentiation. It can transcriptionally regulate the expression of placental lactogen I and proliferin in mouse placental trophoblast giant cells [37, 38]. Moreover, GATA3 can interact with the placental transcription factor GCM1 to inhibit its activity and suppress trophoblast invasion [39]. In addition, GATA3 can promote extraembryonic trophoblast fate at the blastocyst stage and drive trophoblast differentiation in trophoblast stem cells [32]. Our study indicates that hypoxia‐induced O‐GlcNAcylation stabilizes GATA3. Therefore, it is possible that hypoxia induces abnormal differentiation and function of human trophoblast cells by inducing O‐GlcNAcylation and stabilizing GATA3 at the maternal–fetal interface during early pregnancy. The dysregulation of various GATA3 functions and their effects on multiple gestational processes may explain the molecular basis of multiple dysfunctions in E‐PE placentas.

O‐GlcNAcylation is a multifunctional posttranslational modification that regulates protein function [40]. However, its role in placental development has not been well studied. We previously identified O‐GlcNAcylated proteins from human placenta and found that GATA3 was significantly upregulated for O‐GlcNAcylation during trophoblast syncytialization [22]. Hypoxia induces the O‐GlcNAcylation of GATA3 and increases its stability. However, the mechanism underlying this process remains unknown. It has been reported that GATA3 binds to the E3 ligase Fbw7, which mediates the ubiquitination degradation of GATA3 [41]. The binding of Fbw7 to GATA3 is enhanced by phosphorylation at Thr^156^ of GATA3, which promotes ubiquitination degradation [41]. We speculated that Thr^322^ O‐GlcNAcylation of GATA3 may induce structural changes that prevent the binding of GATA3 to Fbw7, thereby inhibiting the degradation of GATA3. Further studies are needed to elucidate the mechanism by which O‐GlcNAc modification affects the stability of GATA3.

The syncytiotrophoblast, the primary site of steroid hormone synthesis in the placenta, is formed through cell fusion of the mononucleated cytotrophoblasts. This process is primarily triggered by the activation of protein kinase A (PKA) signal pathway [42]. Protein O‐GlcNAcylation is facilitated by the HBP [40]. It is well established that glutamine fructose‐6‐phosphate amidotransferase (GFAT), the rate‐limiting enzyme of HBP that catalyzes the conversion of fructose‐6‐phosphate to glucosamine‐6‐phosphate, relies on the cAMP/PKA pathway [43]. Hypoxia has been recognized as a significant contributor to PE [12]. Hypoxia can markedly modify the O‐GlcNAc modification of proteins by inducing changes via the stimulation of the HBP flux [13, 14]. In our previous study, using quantitative O‐GlcNAc proteomics, we established a database of O‐GlcNAcylated proteins in human placental trophoblasts [22]. We identified hundreds of proteins that were dynamically O‐GlcNAcylated during trophoblast differentiation, with GATA3 being one of the most significantly changed. Based on these findings, we propose that hypoxia plays a crucial role in regulating T_0_ synthesis by GATA3. Additionally, alterations in glucose and lipid levels can affect the level of O‐GlcNAc modification [44, 45], and may also affect GATA3 stability and thus T_0_ synthesis. Indeed, glucose and lipid metabolism have been reported to be dysregulated in adverse pregnancy outcomes such as PE [46, 47, 48, 49, 50, 51]. However, due to limited investigation into the mechanism responsible for aberrant T_0_ production in PE placentas, no additional evidence has been found so far. A comprehensive study is necessary to fully elucidate the regulatory mechanism that governs GATA3 stability and T_0_ synthesis.

The present study had some limitations. Firstly, it solely involved in vitro experiments, no in vivo studies were conducted to validate the findings of these in vitro tests. This is due to the fact that sex hormone synthesis in pregnant mice predominantly occurs in the ovarian corpus luteum rather than the placenta [52]. Therefore, despite the extensive use of the murine model for investigating reproductive pathophysiology owing to its irreplaceable advantages, it may not be appropriate for studying placental endocrine function [53]. Secondly, the sample size was relatively small. Future studies should aim to increase participant numbers and establish non‐human primate models of preeclampsia placental endocrine disorders to fully elucidate the regulatory mechanisms that maintain placental hormone homeostasis.

## 4 Conclusions

In conclusion, this study provides a novel insight into the regulation of endocrine homeostasis by revealing that hypoxia‐induced O‐GlcNAcylation of GATA3 in placental trophoblasts not only stabilized GATA3 protein but also enhanced T_0_ production. These results suggest a potential mechanism for excessive androgen production that contributes to placental endocrine dysregulation observed in E‐PE. Further investigation is required to fully elucidate the cause of excessive androgen production in E‐PE placentas, which may lead to the development of novel therapeutic strategies for adverse pregnancy outcomes such as E‐PE.

## 5 Materials and Methods

### 5.1 Study Participants and Samples

The experiments were conducted in accordance with the 1964 Declaration of Helsinki and its later amendments [54]. The enrolled participants were pregnant Chinese Han women who underwent regular prenatal care at the Peking University Third Hospital. Pregnancy outcomes were determined according to the definitions in Williams Obstetrics (25th edition) and the American College of Obstetricians and Gynecologists (ACOG) guidelines [55]. In brief, PE was identified in pregnant women who had no history of preexisting or chronic hypertension but demonstrated a systolic blood pressure ≥140 mmHg or diastolic blood pressure ≥90 mmHg on at least two occasions, accompanied by proteinuria (≥ 0.3 g/24 h or ≥(+) in random urine sample); or no proteinuria, but with multiple organ damage after the 20th week of gestation. Based on whether the onset of clinical signs was earlier than the 34th week, PE was further classified as E‐PE or late‐onset PE (L‐PE). Given that the gestational duration in E‐PE is often earlier than term, placentas from unexplained preterm labor were collected as gestational age‐matched controls (CTRL) for E‐PE [56]. Unexplained preterm labor was defined as the onset of labor earlier than 37 weeks with an unknown etiology and no other complications. Pregnancies complicated by gestational diabetes, renal or cardiovascular disease, intrauterine fetal death, fetal chromosomal or congenital abnormalities, or those conceived using assisted reproductive technologies were excluded from the study. 6 E‐PE placentas and 6 CTRL placentas were collected within 1 h of caesarean section and biopsies of the basal plate were collected from the placental disc at sites 5 cm from the umbilical cord insertion site. The samples were either snap‐frozen in liquid nitrogen or fixed in paraformaldehyde before being embedded in paraffin. The clinical characteristics of the pregnant women are summarized in Table S1.

### 5.2 Single‐Cell RNA‐Seq Data Analysis

Violin‐based single‐cell transcriptome sequencing data, from Tsang et al. [28], were aligned and quantified using the Cell Ranger Single‐Cell Software Suite (version 3.0, 10× Genomics, Pleasanton, CA, USA) [57] against the GRCh38 human reference genome. Cells with fewer than 500 detected genes, mitochondrial genes, genes that were expressed in fewer than three cells, and for which the total mitochondrial gene expression exceeded 20% were removed. The R package Seurat (version 2.3.3) [58] was used for normalization, shared nearest‐neighbor graph‐based clustering, differential expression analysis, and visualization.

### 5.3 Immunoprecipitation (IP)

Placental tissues or cultured cells were homogenized with lysis buffer to extract total proteins, and protein concentrations were measured using a BCA Protein Assay Kit (Beyotime Biotechnology). Specific antibodies (anti‐GATA3, anti‐immunoglobulin G [IgG], or anti‐His) and 20 µL agarose beads (Sigma‐Aldrich) were added to 500 µg total proteins and agitated overnight at 4°C. The beads were washed with PBS (phosphate buffer saline, pH = 7.2) by centrifugation at 3000 × *g* and 4°C, and the bound proteins were separated using SDS‐PAGE (sodium dodecyl sulfate polyacrylamide gel electrophoresis) with subsequent immunoblotting analysis. Primary antibodies are listed in Table S2.

### 5.4 Chromatin Immunoprecipitation (ChIP)

A ChIP assay was performed using an EZ‐ChIP^TM^ Kit (Millipore) according to the manufacturer's instructions, as previously described [59, 60]. Briefly, cultured cells were fixed with 1% formaldehyde and glycine was added to terminate the reaction. The cells were harvested in PBS buffer containing a protease inhibitor cocktail (Millipore), and the extracted DNA was degraded to ∼300 bp fragments in an ultrasonic cell disruptor. The DNA fragments were immunoprecipitated with anti‐GATA3 antibody or mouse IgG (negative control) to obtain GATA3 protein–DNA complexes, which were subsequently reverse cross‐linked. Following DNA purification and elution, qPCR was performed to measure the level of *HSD3B1* and *HSD17B3*. Specific primers for *HSD3B1* and *HSD17B3* are listed in Table S3.

### 5.5 Plasmid Construction and Transfection

Full‐length GATA3 cDNA (sequence obtained from the Human ORFeome Database, http://horfdb.dfci.harvard.edu/) was amplified using PCR and subcloned into the pcDNA4/myc‐His vector (WT‐G). PCR‐based site‐directed mutagenesis plasmids were constructed using TransStart FastPFU DNA polymerase (TransGen Biotech, Beijing, China), with the WT‐G plasmid as the template. Serine (S^316^, S^369^, S^370^) or threonine (T^315^, T^322^) of GATA3 was mutated to alanine and named S^316A^‐G, S^369A^‐G, S^370A^‐G, T^315A^‐G, and T^322A^‐G, respectively. The construct sequences were validated by DNA sequencing. The primers used for PCR amplification are listed in Table S4.

Plasmid transfection experiments were performed as previously described [61]. Briefly, the trophoblast cell line JEG3 was cultured in 6‐well plates in DMEM (Dulbecco's modified Eagle's medium) supplemented with 10% fetal bovine serum and antibiotics. At 60–70% confluency, 1 µg specific plasmid mixed with lipofectamine 2000 (Invitrogen, Shanghai, China) were added. The cells were maintained in an incubator at 37°C before harvesting.

### 5.6 Dual‐Luciferase Reporter Assay

The PCR amplicon containing the GATA3‐binding sequence at the human *HSD3B1* promoter was cloned into the pGL3‐promoter luciferase vector to generate the pGL3‐HSD3B1‐promoter plasmid (3B1‐P), as previously described [26]. Different lengths of sequences containing the predicted GATA‐binding sequence (22 kb downstream of the transcription start site of the *HSD17B3* gene) were PCR amplified (primers shown in Table S4) and subcloned into the pGL3‐promoter–enhancer plasmid to generate the plasmids pGL3‐HSD17B3‐enhancer 1 (17B3‐E1), pGL3‐HSD17B3‐enhancer 2 (17B3‐E2), and pGL3‐HSD17B3‐enhancer 3 (17B3‐E3).

The dual‐luciferase reporter assay was performed as previously described [26]. Briefly, JEG3 cells were grown to 80% confluence in 24‐well plates (approximately 1 × 10^5^ cells/well) and transfected with plasmid 3B1‐P, 17B3‐E1, 17B3‐E2, or 17B3‐E3 with or without plasmid WT‐G. After 48 h of transfection, the cells were harvested using a passive lysis buffer and luciferase activity was measured using a Dual‐Luciferase Reporter Assay Kit (Promega, Madison, WI, USA) and a luminometer (GloMax, Promega), according to the manufacturer's instructions. *Renilla* luciferase activity was used to normalize the *firefly* luciferase activity.

### 5.7 Measurement of T_0_

The placental tissues were homogenized in PBS with a Bead Ruptor 24 Elite (OMNI International, Beijing, China), and the supernatants were collected after centrifugation (10,000 × *g* for 10 min) at 4°C. The total T_0_ level in the placental tissue or cultured cells was measured using a Siemens IMMULITE 2000 immunoassay system (Siemens, Munich, Germany) according to the manufacturer's instructions.

### 5.8 Heatmap Analysis

Published datasets (GSE159480) from hypoxia‐exposed induced pluripotent stem cells (iPSCs) [32] were applied in an HBP‐related gene heatmap analysis based on gene FPKM (Fragments Per Kilobase Million). The heatmap display was based on the Z‐score value of gene expression.

### 5.9 Statistical Analysis

All statistical analyses were performed using GraphPad Prism software (version 9.0; GraphPad Software, San Diego, CA, USA). Statistical comparisons between groups were performed using an independent sample nonparametric *t*‐test or unpaired one‐way analysis of variance (ANOVA) for normally distributed data, followed by a post hoc Tukey–Kramer multiple comparison analysis. A nonparametric Spearman's correlation test was used to analyze the correlations between 3β‐HSD1, 17β‐HSD3, GATA3, and T_0_ levels. Results are shown as the mean ± SEM of at least three independent experiments. Statistical significance was set at *p* < 0.05.

Expanded Methods are described in the Supporting Information.

## Author Contributions

J.L. performed the experiments, analyzed the data, prepared figures, and drafted the manuscript. Y.Y. and H.W. participated in IP experiments, data analysis, and figure preparation. Y.X. and W.F. analyzed single‐cell RNA‐seq and ATAC‐seq data. F.D., Y.W., Y.Z., and X.S. collected clinical samples and participated in data analysis. W.Q., Y.Z., and C.W. performed LC‐MS/MS experiment and analyzed the corresponding data. Y‐X.L. assisted with cell culture and Western blotting. Y‐L.W. and X.S. designed and supervised the study, coordinated the research team, and revised the draft. All authors have read and approved the final manuscript.

## Ethics Statement

The protocol for collection of human placental tissue and plasma samples, as well as the animal experiments were approved by the Ethics Committee of the Institute of Zoology, Chinese Academy of Sciences, and Peking University Third Hospital (Beijing, China) with approval numbers IOZ‐YSB‐2022‐02 and IOZ‐YSB‐2021‐14 for clinical and animal experiments, respectively. All pregnant women enrolled in this study signed written informed consent.

## Conflicts of Interest

The authors declare no conflicts of interest.

## Supporting information



Supporting Information

## Data Availability

The datasets used and/or analyzed during the current study are available from the corresponding author on reasonable request.
