# Supplementary material for: Hypoxia‐Induced O‐GlcNAcylation of GATA3 Leads to Excessive Testosterone Production in Preeclamptic Placentas
Source: MedComm (2020). 2025 Feb 23;6(3):e70115. doi: 10.1002/mco2.70115 (PMC11847629; doi:10.1002/mco2.70115)
Supplement: Supplementary file 1 — Supporting Information [file MCO2-6-e70115-s001.docx]

**Supplemental Material for**

**Hypoxia-induced O-GlcNAcylation** **of GATA3 leads to excessive testosterone production in preeclamptic placentas**

Juan Liu^1,^^3,#^, Yun Yang^1,7,#^, Hongyu Wu^1,7,#^, Feihong Dang^1^, Xin Yu^1^, Feiyang Wang^1^, Yongqing Wang^4^, Yangyu Zhao^4^, Xiaoming Shi^4^, Wei Qin^5^, Yanling Zhang^5^, Yu-Xia Li^1^, Chu Wang^5,6^, Xuan Shao^1,^^2,7,*^, Yan-Ling Wang^1,2,7,*^

^#^ These authors contribute equally to this work.

^*^ All correspondence should be addressed to Dr. Yan-Ling Wang at wangyl@ioz.ac.cn or Dr. Xuan Shao at shaoxuan@ioz.ac.cn

**This file includes:**

Expanded methods

Figures S1 to S7

Tables S1 to S4

References

**Expanded Methods**

**Antibodies and Reagents**

The chemicals and culture media components used in this study were purchased from Sigma-Aldrich (St. Louis, MO, USA), unless otherwise specified. A detailed description of the antibodies is provided in Table S2.

**Cell culture**

The human choriocarcinoma cell line JEG3 was purchased from the American Type Culture Collection (ATCC; Manassas, VA, USA). After thawing, the cells were maintained in Dulbecco’s modified Eagle’s medium (DMEM; Hyclone, Logan City, UT, USA) supplemented with 10% FBS (Hyclone) and antibiotics in 5% CO_2_ at 37 °C.

**Western blotting**

Cultured cells or tissues were lysed using RIPA lysis buffer (CWBIO, Beijing, China) supplemented with protease inhibitors (Sigma-Aldrich). The supernatant was collected after centrifugation at 10,000 × *g* for 10 min to remove insoluble fractions. The protein concentration was determined using a BCA Protein Assay Kit (Beyotime Biotechnology, Shanghai, China). 40 µg protein was resolved on 10% SDS-PAGE gels and electrophoretically transferred to a nitrocellulose membrane (Millipore, Boston, MA, USA). The membranes were blocked with 5% bovine serum albumin (BSA) blocking buffer (5% BSA solution and 0.1% Tween 20 in phosphate-buffered saline [PBS]) and incubated with the indicated primary antibodies (Table S2), followed by incubation with the corresponding horseradish peroxidase (HRP)-conjugated secondary antibodies (Jackson, MI, USA). The signals were visualized using an ECL kit (Thermo Fisher Scientific, Waltham, MA, USA), captured using an GeneGnome XRQ Chemiluminescence Imaging System (Syngene, Frederick, MD, USA). The images were analyzed using ImageJ software (National Institutes of Health, Bethesda, MD, USA).

**Immunohistochemistry of paraffin section**

Freshly collected human placental tissues were fixed in 4% paraformaldehyde (PFA) (Sigma-Aldrich) overnight at 4 °C, dehydrated in serial concentrations of ethanol, cleared in xylene, and embedded in paraffin wax. Paraffin sections (5 μm thick) were routinely deparaffinized in xylene, rehydrated in serial concentrations of ethanol, antigen retrieved, and blocked before incubation with a specific antibody against GATA3, 3β-HSD1, or 17β-HSD3 (Table S2). Incubation with species-specific IgG served as the negative control. Following incubation with horseradish peroxidase (HRP)-conjugated specific secondary antibodies (Zhong Shan Golden Bridge, Beijing, China) for 1 h at room temperature, signals were visualized using DAB solution (Dako Cytomation, Glostrup, Denmark). Sections were counterstained with hematoxylin and mounted. Images were captured using a light microscope (DP72, Olympus, Osaka, Japan).

**RNA extraction and real-time quantitative polymerase chain reaction (****RT-qPCR)**

Total RNA was extracted from cultured cells using TRIzol reagent (Invitrogen). RNA concentration was quantified using a NanoDrop spectrophotometer (Thermo Fisher Scientific). Reverse transcription of 2 µg total RNA was performed using M-MLV Reverse Transcriptase (Promega, Madison, WI, USA) according to the manufacturer’s instructions. RT-qPCR was performed using SYBR Premix ER Taq II (Takara, Osaka, Japan), and the reaction was carried out at 95 °C for 30 s, followed by 40 cycles of 95 °C for 5 s and 60 °C for 31 s. The primer sequences are summarized in Table S3. All reactions were performed in triplicate, the fold change of gene expression was calculated using the 2^-△△CT^ method and normalized to the expression of *β-actin*.

**Identification of GATA3 O-GlcNAcylation sites**

Total protein from JEG3 cells was extracted as described above, and immunoprecipitated GATA3 was eluted by boiling the beads in loading buffer and then resolved by SDS-PAGE. The gel slice of the GATA3 band at the appropriate position was collected, washed with Milli-Q water, destained with a 1:1 solution of 50 mM ammonium bicarbonate (ABC)/acetonitrile for 30 min, and then dehydrated in 100% acetonitrile. The gel slices were rehydrated with 10 mM DTT in 50 mM ABC and incubated for 45 min at 56 °C to reduce thiols. The slices were subsequently incubated with 55 mM iodoacetamide in 50 mM ABC in the dark at room temperature and the treated gel slices were dehydrated in 100% acetonitrile. Gel pieces were rehydrated in a trypsin solution (2 ng/μL) and incubated at 37 °C for 16 h. Peptides were eluted in 50% acetonitrile with 5% (v/v) TFA and dried using a SpeedVac (Thermo Fisher Scientific). Finally, the resulting peptides were subjected to LC–MS/MS on an Easy-nLC 1000 UPLC (Thermo Fisher Scientific) coupled with a Q Exactive Orbitrap mass spectrometer. The spectra were analyzed using Mascot (version 2.2; Matrix Science, London, UK) with a differential modification of 203.079373 Da to obtain O-GlcNAcylated peptides, which were manually verified to locate the O-GlcNAcylated site.

**Single cell ATAC-seq (assay for transposase-accessible chromatin using sequencing) data analysis**

Single cell chromatin accessibility analysis was performed using the publicly available single-cell ATAC-seq and RNA-seq datasets ^1,2^. R package Signac (version 1.14.0) was used for chromatin dynamics, peak normalization, and clustering functions analysis ^3^.

**Culture of primary human trophoblast (PHT) cells from human term placenta**

PHT cells were isolated from human placenta as previously described ^4^. In brief, minced placental tissues were digested with trypsin (Sigma-Aldrich, MO, USA) and DNase I (Sigma-Aldrich). The supernatant was subsequently centrifuged, and the resulting cell suspension was separated by a Percoll density gradient (GE Healthcare BioSciences AB, Uppsala, Sweden). PHT cells were then collected from the 30-50% density layer and cultured in Ham’s F12: DMEM medium (HyClone, CT, USA) supplemented with 10% fetal bovine serum (FBS) and antibiotics in a 5% CO_2_ air incubator at 37 °C. Unattached cell debris was removed approximately 10 h after seeding, and the cells were treated with TMG/DMSO or exposed to hypoxia (2% O_2_)/ normoxia (21% O_2_). Cells or culture supernatant was collected according to experimental requirements.


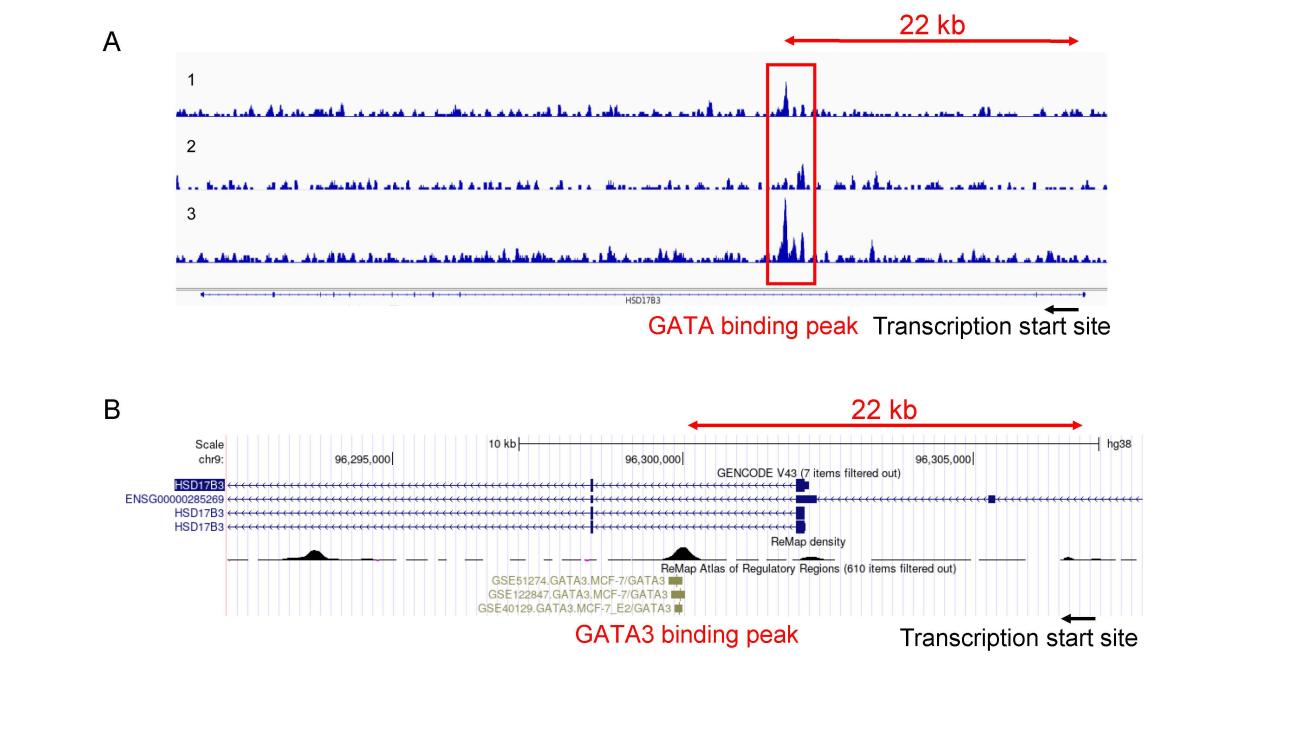


**Figure S1. Potential GATA-binding motifs in the *HSD17B3* gene locus.** (**A**) The putative GATA-binding motif (red frame) is located approximately 22 kb downstream of the transcription start site (TSS) of *HSD17B3* gene. The data was obtained from the ChIP-seq database (GSE105081) on the Integrative Genomics Viewer website. The numbers on the left are samples from different individuals in the ChIP-seq database. (**B**) The hg38 genome was chosen from the transcription factor database, as sourced from the UCSC database (http://genome.ucsc.edu/). Target gene was inputted, and gene interval determined. The binding region of the GATA3 transcription factor, upstream of the *HSD17B3* gene, was then predicted. To predict the binding sites of the GATA3 transcription factor, the ReMap ChIP-seq database was selected within the regulation option.

**A**


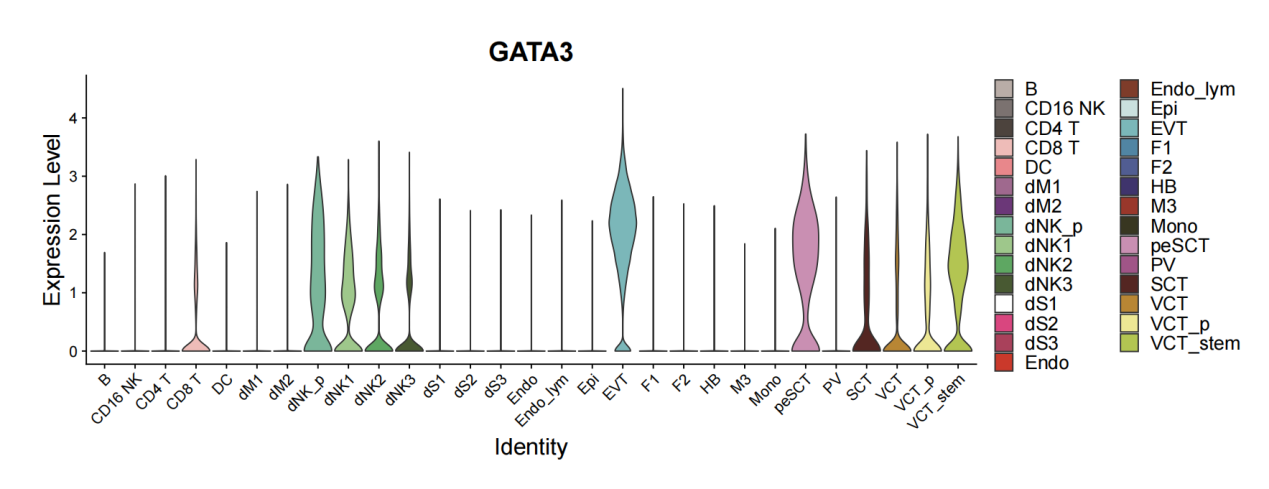


**B**


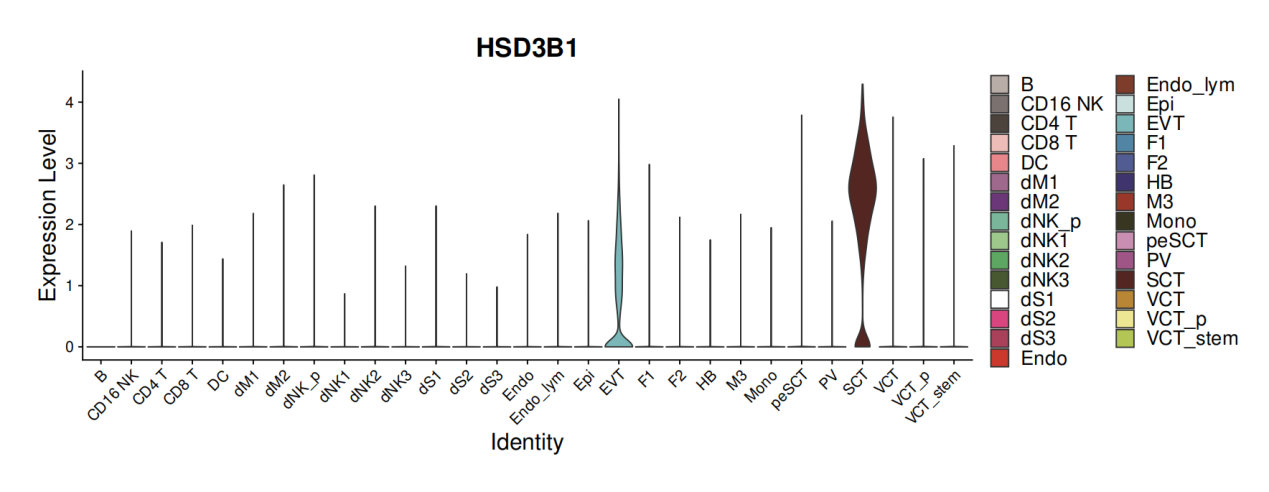


**C**


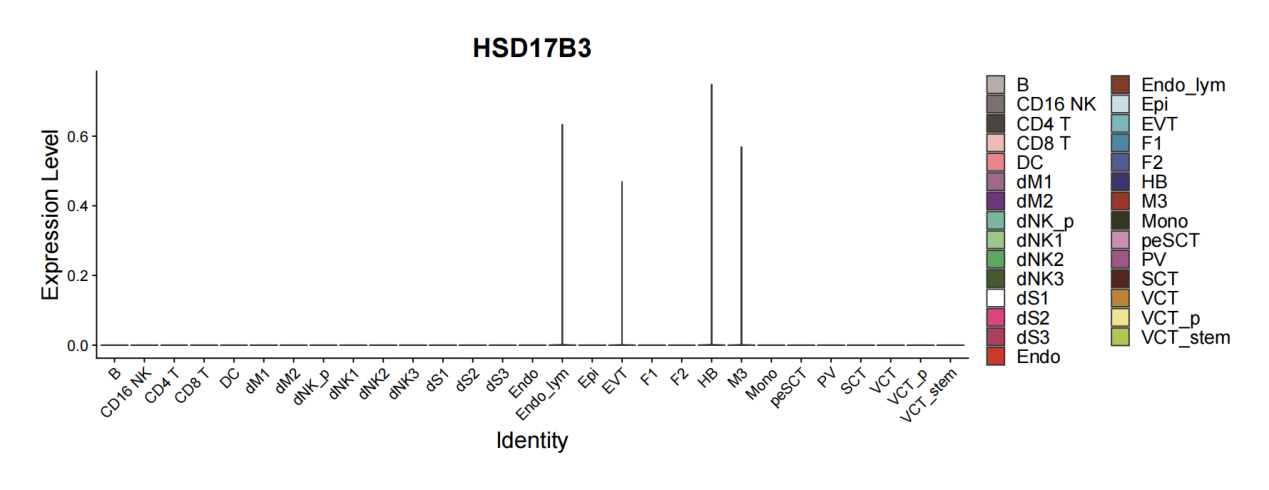


**Figure S2. Cell type-specific expression of *GATA3*, *HSD3B1*, and *HSD17B3* in normal human placenta.** (**A-C**) Single-cell transcriptome sequencing of *GATA3* (**A**), *HSD3B1* (**B**), and *HSD17B3* (**C**) expression across various cell types in normal human placenta, including immune cells, trophoblast cells, and others. B, peripheral B cell; CD16 NK, CD16^+^ natural killer cell; CD4 T, CD4^+^ T cell; CD8 T, CD8^+^ T cell; DC, dendritic cell; dM, decidual macrophage; dNK, decidual natural killer cell; dNK_p, proliferative decidual natural killer cell; dS, decidual stromal cell; Endo, endothelial cell; Endo_lym, lymphatic endothelial cell; Epi, epithelial glandular cell; EVT, extravillous trophoblast; F, fibroblast; HB, Hofbauer cell; Mono, monocyte; preSCT, pre-fusion syncytiotrophoblast; PV, perivascular cell; SCT, syncytiotrophoblast; VCT, villous cytotrophoblast; VCT_p, proliferative villous cytotrophoblast; VCT_stem, stem villous cytotrophoblast.


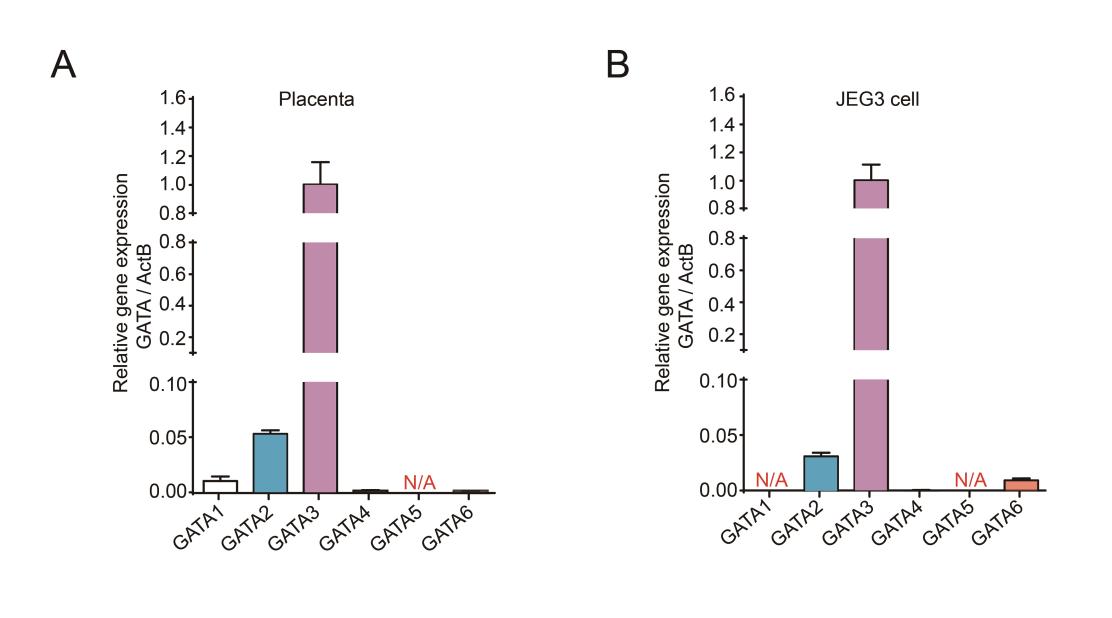


**Figure S3.** **Panorama expression of GATA family members in placental tissue and trophoblast cells.** (**A**-**B**) mRNA expression of GATA family members in normal placentas (**A**) and JEG3 cells (**B**). Data are shown as the mean ± SEM of at least three independent experiments and were analyzed by one-way ANOVA and Tukey–Kramer multiple comparison test.


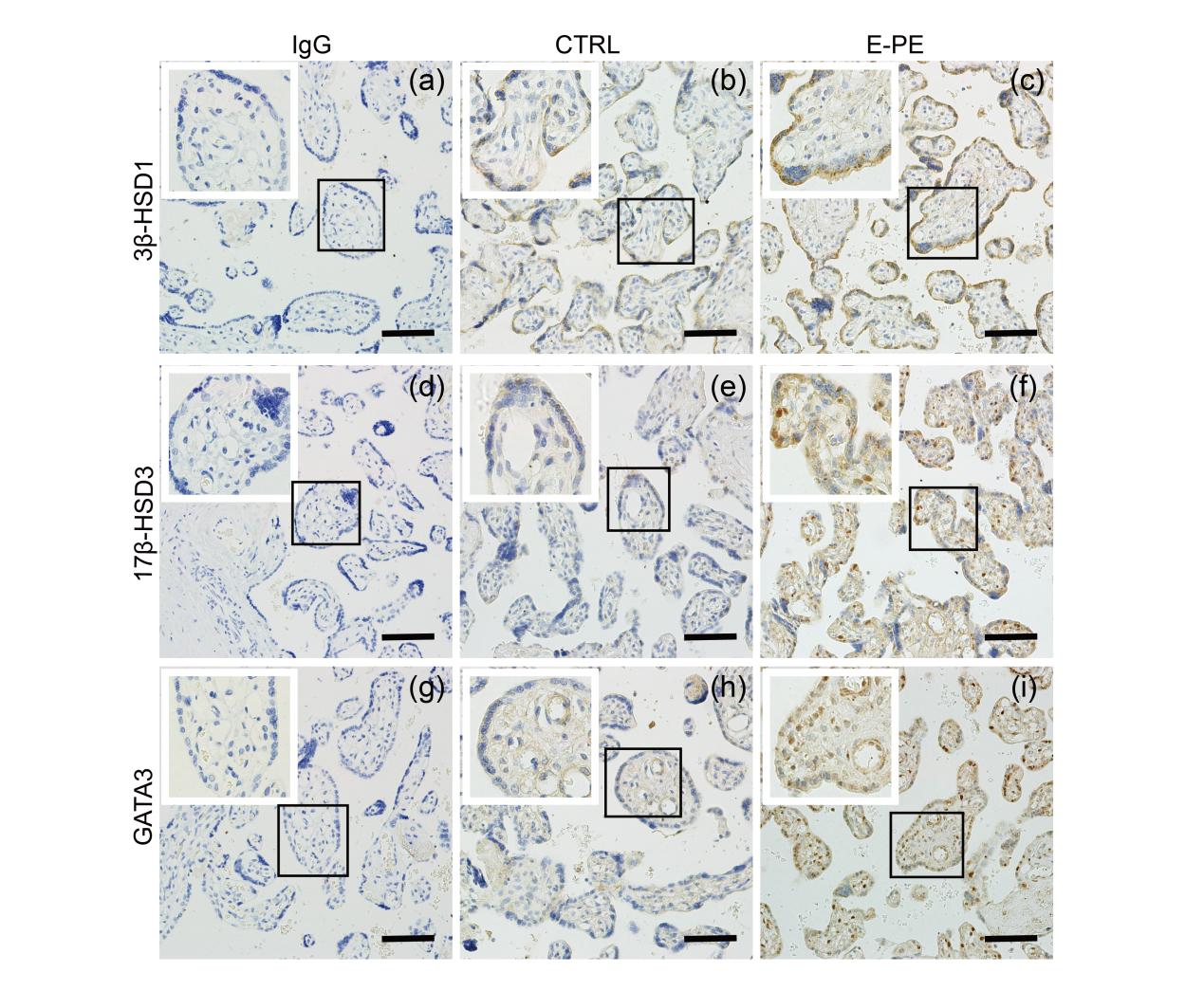


**Figure S4.** **Immunohistochemistry of 3β-HSD1, 17β-HSD3, and GATA3 in E-PE and** **CTRL placentas.** The insert, depicted in white-box format, represents a magnified view of the region delineated in black-box format. Scale bar, 50 μm.


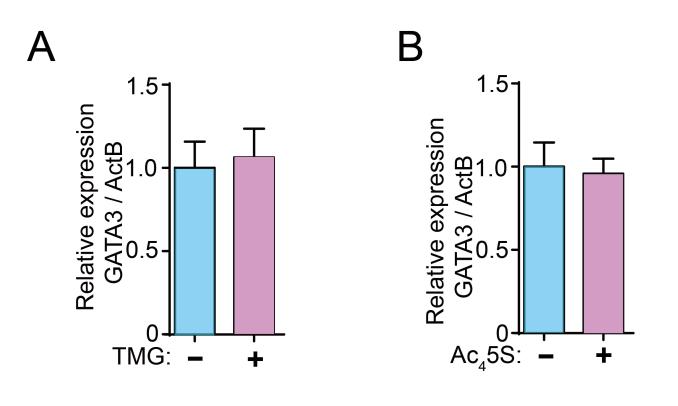


**Figure S5. *GATA3* expression after TMG and Ac_4_5S treatment in JEG3 cells.** (**A**-**B**) mRNA level of *GATA3* in JEG3 cells treated with 20 μM TMG (**A**) or Ac_4_5S (**B**) for 24 h. Data are shown as the mean ± SEM of at least three independent experiments and were analyzed by a two-tailed *t*-test.

**A**


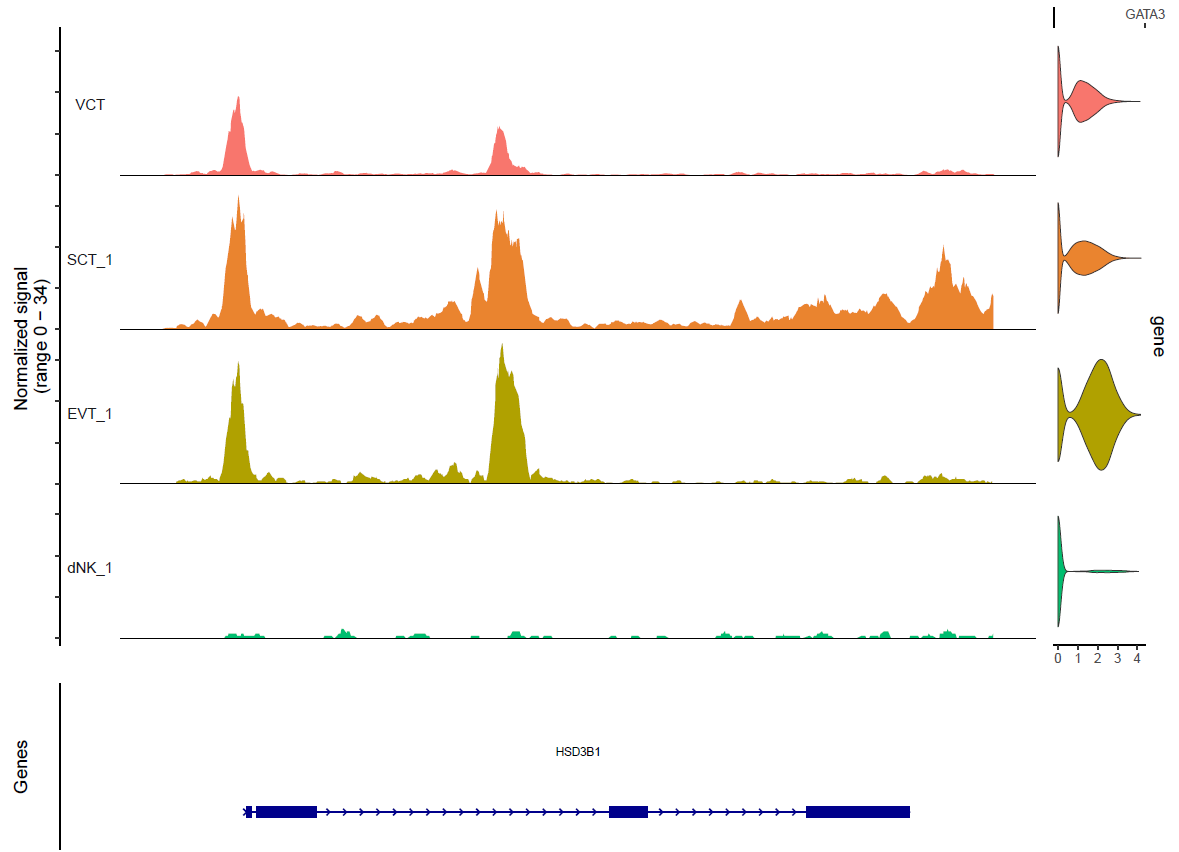


**B**


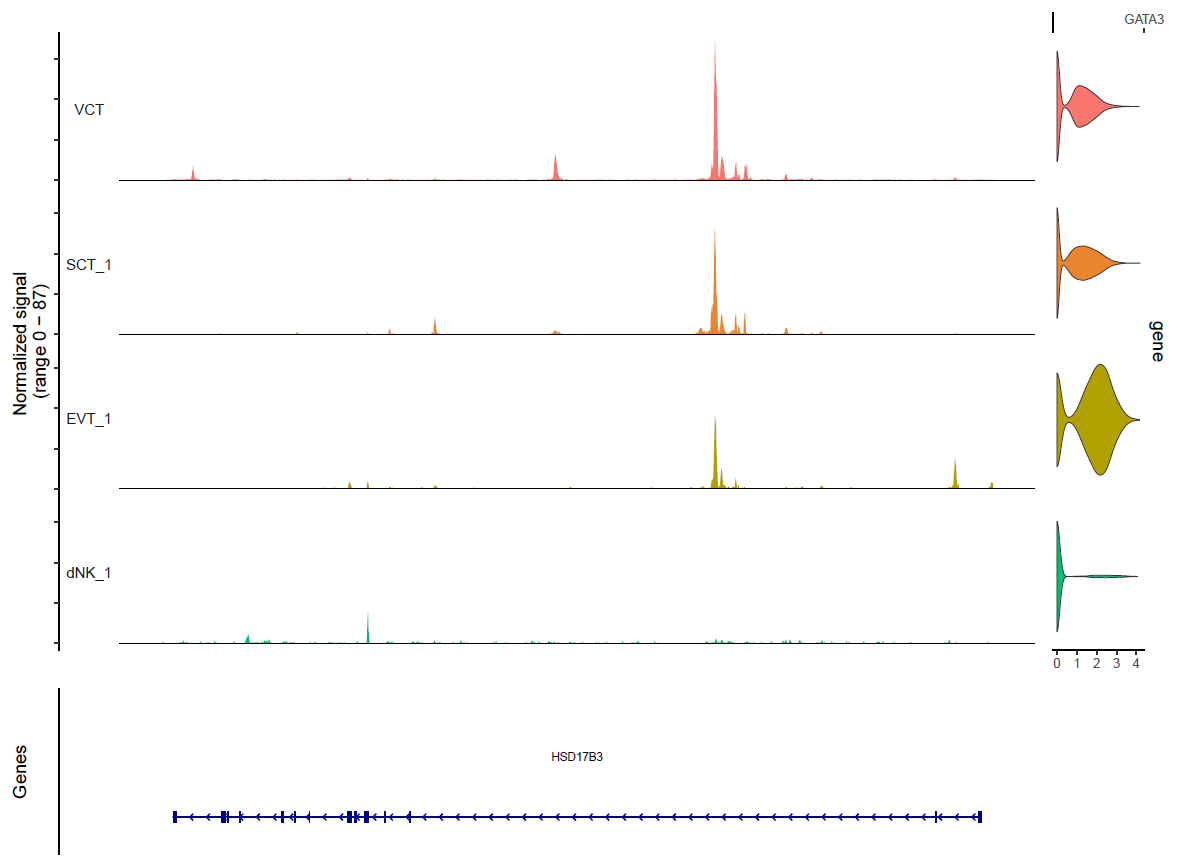


**Figure S6. Single-cell ATAC-seq analysis of *HSD3B1* and *HSD17B3*.** (**A**-**B**) Single-cell ATAC-seq analysis revealed the chromatin accessibility of *HSD3B1* (**A**) and *HSD17B3* (**B**) gene locus in four cell types (VCT, SCT, EVT, and dNK). *GATA3* expression is also shown in these cells.


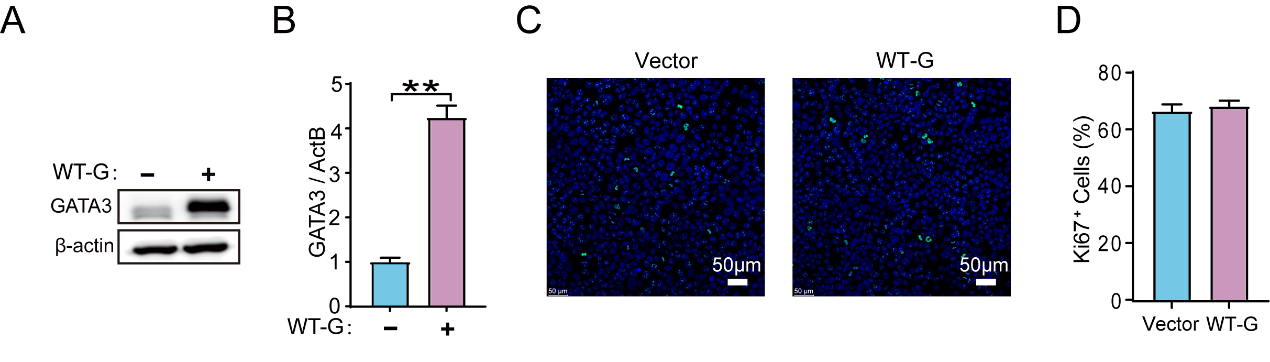


**Figure S7. GATA3 overexpression does not influence JEG3 cells proliferation.** (**A**-**B**) Western blotting (**A**) and statistical analysis (**B**) of GATA3 in JEG3 cells transfected with either WT-G or Vector plasmid. (**C**-**D**) Representative images of immunofluorescence staining for Ki67 (**C**) and statistical analysis of the proportion of cells expressing Ki67 (**D**) in JEG3 cells transfected with either WT-G or Vector plasmid. Immunofluorescence staining was performed on three slides per group. The proportion of Ki67^+^ cells was determined by counting five random fields from each slide and presented as the ratio of Ki67^+^ cell number to total cell number. Data are shown as the mean ± SEM of at least three independent experiments and were analyzed by two-tailed *t*-test. **, *P* < 0.01.

| **Table S1.** Clinical characteristics of the pregnant women enrolled in this study | | |
| --- | --- | --- |
|  | Preterm labor  (n=6) | Early-onset Preeclampsia  (n=6) |
| Maternal Age (years) | 29.1 ± 1.2 | 28.8 ± 3.3 |
| BMI (kg/m^2^) | 21.3 ± 4.9 | 22.0 ± 5.8 |
| Systolic Blood Pressure (mmHg) | 116.8 ± 8.4 | 165.8 ± 7.3* |
| Diastolic Blood Pressure (mmHg) | 79.3 ± 7.1 | 99.1 ± 8.9* |
| 50g Glucose Challenge Test (mmol/L) | 6.5 ± 1.3 | 7.0 ± 1.5 |
| Urine Protein (g/24h) | - | 3.8 ± 2.3* |
| Gestational Week (w) | 31.1 ± 4.0 | 30.9 ± 3.7 |
| Infant Birth Weight (g) | 2296 ± 595 | 1806 ± 415* |
| Data are presented as mean ± SD, and significant difference between groups was analyzed by Student’s t-test. *Compared with preterm labor, p<0.05. BMI, body mass index: weight (kg) divided by the square of the height (m). | | |

| **Table S2.** List of antibodies used in this study | | | |  | |  |
| --- | --- | --- | --- | --- | --- | --- |
| Peptide/protein target | Name of Antibody | Manufacturer, catalog # | Species | | RRID | Dilution used |
| O-GlcNAc | anti-O-GlcNAc antibody | #9875 | mouse | | AB_10950973 | 1:1000 |
| β-actin | anti-β-actin antibody | ab8227 | rabbit | | AB_2305186 | 1:5000 |
| GATA3 | Anti-GATA3 antibody | MA1-028 | mouse | | AB_2536713 | 1:1000 |
| 3β-HSD1 | Anti-3β-HSD1 antibody | ab55268 | mouse | | AB_942015 | 1:1000 |
| 17β-HSD3 | Anti-17β-HSD3 antibody | ab102771 | rabbit | | AB_10711924 | 1:1000 |
| 6× His tag | Anti-6× His tag antibody | ab9108 | rabbit | | AB_307016 | 1:2000 |
| Ki67 | Ki-67 (D3B5) | 9129T | rabbit | | AB_2687446 | 1:800 |
| Secondary antibody | FITC-labeled Anti-Rabbit  IgG (H+L) | ZF-0311 | goat | | AB_2571576 | 1:300 |
| Secondary antibody | Anti-Rabbit IgG (H+L) | 111-035-003 | goat | | AB_2313567 | 1:8000 |
| Secondary antibody | Anti-Mouse IgG (H+L) | 111-005-003 | goat | | AB_2338447 | 1:8000 |
| Secondary antibody | Rabbit two-step detection kit | PV-9001 | goat | | AB_2868452 | N/C |
| Secondary antibody | mouse two-step detection kit | PV-9002 | goat | | AB_2904194 | N/C |
| Secondary antibody | VeriBlot for IP Detection Reagent (HRP) | ab131366 | N/C | | AB_2892718 | 1:500 |

**Table S3. Sequence of the primers used for real-time qPCR in this study.**

| Gene name | Primers | Sequence（5'~3'） |
| --- | --- | --- |
| *GATA1* | Forward primer  Reverse primer | TGCGGCCTCTATCACAAGATG  CTGCCCGTTTACTGACAATCA |
| *GATA2* | Forward primer  Reverse primer | ACTGACGGAGAGCATGAAGAT  CCGGCACATAGGAGGGGTA |
| *GATA3* | Forward primer  Reverse primer | GCCCCTCATTAAGCCCAAG  TTGTGGTGGTCTGACAGTTCG |
| *GATA4* | Forward primer  Reverse primer | GTGTCCCAGACGTTCTCAGTC  GGGAGACGCATAGCCTTGT |
| *GATA5* | Forward primer  Reverse primer | CTTCGTGTCCGACTTCTTGGA  CCGAGGCATTCCTTGTGGA |
| *GATA6* | Forward primer  Reverse primer | CTCAGTTCCTACGCTTCGCAT  GTCGAGGTCAGTGAACAGCA |
| *HSD17B3* | Forward primer  Reverse primer | AAAGGCAAAACAACTCAAAGGG  CTCTCTAAAGCCTGCTACCTG |
| *HSD3B1* | Forward primer  Reverse primer | TCCCATAGGAGGAGAGAGCA  CCTCATTTCCTGTGGCAAGT |
| *ActB* | Forward primer  Reverse primer | CGAGCACAGAGCCTCGCCTT  TGCACATGCCGGAGCCGTTG |

**Table S4. Sequence of the primers used for plasmids construction in this study.**

| plasmid name | Primers | Sequence（5'~3'） |
| --- | --- | --- |
| WT-GATA3 | Forward primer  Reverse primer | GCTCTAGAATGGAGGTGACGGCGGACCAG  GGAATTCCTAACCCATGGCGGTGACCAT |
| T^315A^-GATA3 | Forward primer | gcaggggcgtcctgtgcGAAC-  TGTCAGACCACCACAACCAC  GCACAGGACGCCCCTGCTCTC-  CTGGCTGCAGACAGCCTTCG |
|  | Reverse primer |  |
| S^316A^-GATA3 | Forward primer | AGGGACGCGGTGTGCGAACTG-  TCAGACCACCACAACCACAC  CGCACACCGCGTCCCTGCTCT-  CCTGGCTGCAGACAGCCTTC |
|  | Reverse primer |  |
| T^322A^-GATA3 | Forward primer | CTGTCAGGCCACCACAACCAC-  ACTCTGGAGGAGGAATGCCAAT  GGTTGTGGTGGCCTGACAGTTC-  GCACAGGACGTCCCTGCTC |
|  | Reverse primer |  |
| S^369A^-GATA3 | Forward primer | TGGCTAGCAAATCCAAAAAGTG-CAAAAAAGTGCATGACTCACTGG |
|  | Reverse primer | TTGGATTTGCTAGCCATTTTTCGG-TTTCTGGTCTGGATGCCTTCC |
|  |  |  |
| S^370A^-GATA3 | Forward primer | TGTCTGCCAAATCCAAAAAGTGC-  AAAAAAGTGCATGACTCACTGG  TTGGATTTGGCAGACATTTTTCGG-  TTTCTGGTCTGGATGCCTTCCTT |
|  | Reverse primer |  |
| HSD17B3-E1 | Forward primer | TTGGTACCCCAGAAAGGCAAAACAACTCA |
|  | Reverse primer | TTACTCGAGCATCACAGACAGCAGGC |
| HSD17B3-E2 | Forward primer | TTGGTACCCCAGAAAGGCAAAACAACTCA |
|  | Reverse primer | TTACTCGAGATGGTCTGTAGAGAATCCCCT |

**Table S4. Sequence of the primers used for plasmids construction in this study (continued).**

| plasmid name | Primers | Sequence（5'~3'） |
| --- | --- | --- |
| HSD17B3-E3 | Forward primer | TTGGTACCCCAAATCACAGTTCAAGGC |
|  | Reverse primer | TTACTCGAGATGGTCTGTAGAGAATCCCCT |
| HSD3B1-P | Forward primer | CCGGTACCGACAGCTGGTATCAACTGACT |
|  | Reverse primer | CCGCTCGAGTGATCCTCTGTCCCAGAAAC |

**References**

1. Arutyunyan A, Roberts K, Troule K, et al. Spatial multiomics map of trophoblast development in early pregnancy. *Nature*. Apr 2023;616(7955):143-151.

2. Vento-Tormo R, Efremova M, Botting RA, et al. Single-cell reconstruction of the early maternal-fetal interface in humans. *Nature*. Nov 2018;563(7731):347-353.

3. Stuart T, Srivastava A, Madad S, Lareau CA, Satija R. Single-cell chromatin state analysis with Signac. *Nat Methods*. Nov 2021;18(11):1333-1341.

4. Shao X, Yang Y, Liu Y, et al. Orchestrated feedback regulation between melatonin and sex hormones involving GPER1-PKA-CREB signaling in the placenta. *J Pineal Res*. Dec 2023;75(4):e12913.
